# Genomic characterization of *Bacillus velezensis* BP5 and BP103: insights into biocontrol potential against bacterial leaf spot on pepper

**DOI:** 10.3389/fmicb.2026.1757903

**Published:** 2026-07-21

**Authors:** Le Uyen Thanh, Vu Nhat Tan, Tran Thi Ngoc Huyen, To Lan Phuong, Huynh Ngoc Tam, Luu Phuc Loi

**Affiliations:** 1 Laboratory Biotechnoly, Faculty of Engineering and Technologies, Dong Thap University, Dong Thap, Vietnam; 2 Application Laboratory, TBR Technology Corporation, Ho Chi Minh City, Vietnam; 3 Le Vu Hung Resources Center, Dong Thap University, Dong Thap, Vietnam; 4 Institute for Applied Research in Health Sciences and Aging (ARiHA)-Thong Nhat Hospital, Ho Chi Minh City, Vietnam

**Keywords:** antagonistic activity, *Bacillus velezensis*, carbohydrate-active enzymes, pangenome, secondary metabolites, whole-genome sequencing

## Abstract

*Bacillus velezensis* strains BP5 and BP103, isolated from pepper rhizosphere soil in the Mekong Delta, Vietnam, displayed potent antagonistic activity against *Xanthomonas euvesicatoria*, which causes pepper bacterial spot. In dual-culture assays, BP5 formed the largest inhibition zone (36.7 mm), outperforming BP103 (32.1 mm) and oxolinic acid (31.3 mm). Both Gram-positive, rod-shaped strains produced extracellular protease, lipase, amylase, cellulase, and siderophores. Whole-genome sequencing revealed compact and genetically stable genomes: BP5 (4.08 Mb, 46.04% GC, 4,047 protein-coding genes, 5 prophages) and BP103 (3.91 Mb, 46.46% GC, 3,793 genes, 2 prophages). Phylogenetic reconstruction and average nucleotide identity (ANI of more than 98%) confirmed their assignment to *B. velezensis*, with closest relatedness to strain 160. Orthologous gene analysis across 28 *B. velezensis* genomes and *B. subtilis* DSM10 identified 2,593 single-copy genes. Compared to BP103, BP5 harbors greater numbers of multi-copy orthologs, other orthologs, and unique paralogs (388, 910, and 5 in BP5 with 336, 763, and 1 in BP103, respectively). Pangenome analysis showed that the core genome size decreased progressively with the addition of new genomes; BP5 showed more pronounced genetic divergence driven by expanded unique paralogs and shell gene families (with 456 in BP5 compared with 203 in BP103). Both strains encode 13 secondary metabolite biosynthetic gene clusters, including surfactin, fengycin, bacillaene, macrolactin, difficidin, bacilysin, bacillibactin, mersacidin, and locillomycin, plus four previously undescribed terpene/PKS. The CAZyme repertoire was richest in BP5 (133 genes), exceeding BP103 and the commercial strain FZB42 (both 129 genes), thereby enhancing adhesion and adhesion and rhizosphere colonization. These findings establish BP5 and BP103 as highly promising biocontrol agents, combining high genetic stability with diverse secondary metabolite profiles, suitable for development into sustainable microbial bio-bactericides.

## 1 Introduction

Vietnam is a key pepper producer and exporter in the Asia–Pacific region, ranking seventh in production (Subedi et al., 2024), with the Mekong Delta as the main growing region supplying most pepper for domestic use and export. Although long-term data on global pepper acreage are limited, experimental studies indicate that warming- and drought-like conditions increase disease pressure in pepper, particularly anthracnose, bacterial wilt and, most notably, bacterial spot (BSP), which is expected to negatively affect pepper yield and production in many regions (Shin and Yun, 2010; Jang et al., 2019). BSP can cause yield losses exceeding 50% under unfavorable conditions (Dhakal et al., 2019; Utami et al., 2022), especially during the rainy season, thereby reducing marketable yield, fruit quality and export value. In Vietnam, no biological management strategy for BSP is currently available that is both sustainable and specifically tailored to pepper; therefore, growers rely heavily on chemical pesticides, which increases production costs and promotes resistance development, environmental contamination and health risks. Against this background, the development and deployment of effective biological control agents, such as indigenous *Bacillus* strains antagonistic to *Xanthomonas euvesicatoria* (Milovanović et al., 2025), are urgently needed to support sustainable pepper production in the Mekong Delta.

Plant diseases significantly reduce global crop yields and have accelerated the search for sustainable biological control alternatives to chemical pesticides (Köhl et al., 2019). In soil, the rhizosphere, and internal plant tissues (the endosphere), *Bacillus* is recognized as one of the most common and important bacterial genera. Many species within this genus have been demonstrated to benefit plants, particularly by protecting them against infection by pathogenic agents (Etesami et al., 2023). Among these microbial agents, *B. velezensis* is a Gram-positive, rod-shaped, facultatively anaerobic, endospore-forming bacterium of the *B. subtilis* species complex (Rabbee et al., 2019; Fan et al., 2017). According to Dunlap et al. (2016), this species has been proposed as a heterotypic synonym of *B. amyloliquefaciens* and *B. siamensis* and is treated as an operational group by some authors (Fan et al., 2017). This species excels as a biocontrol agent, promoting plant growth and suppressing pathogens (Fan et al., 2018; Choi et al., 2021; Zhou et al., 2023; Xie et al., 2023).

*B. velezensis* commonly inhabits plant-associated niches, frequently isolated from rhizospheres of ginger, black pepper, and watermelon (Wang K. X. et al., 2022; Wang Q. et al., 2022; Blumenscheit et al., 2022), and as endophytes in rice, olive, coffee, and poplar (Shi et al., 2022; Spantidos et al., 2025; de Castro et al., 2025; Sachin et al., 2021). It excels in root colonization through flagellar motility and biofilm formation, while tolerating abiotic stresses, oxidative pressure, and acidity (Fan et al., 2018; Teixeira et al., 2021; Shen et al., 2023). Furthermore, enzyme secretion that enhances nutrient uptake and plant growth also contributes to plant growth-promoting (PGP) activity (Sibponkrung et al., 2017; Zhang et al., 2024).

Recent studies have confirmed that *Bacillus* represents a rich source of antimicrobial compounds, including volatile compounds, peptides (both ribosomal and non-ribosomal), polyketides, and hybrid metabolites, which are highly effective in the biological control of *Xanthomonas* diseases (Caulier et al., 2019). For example, *Bacillus* strain D13 has been reported to produce 12 volatile compounds, among which decyl alcohol and 3,5,5-trimethylhexanol exhibit strong inhibitory activity against *X. oryzae* pv. *oryzae* in the rice bacterial blight pathosystem (Xie et al., 2018). Moreover, the surfactin-homolog AXLP14 from *B. amyloliquefaciens* B014 demonstrates potent activity against *Xoo* (Li et al., 2016), highlighting the broader potential of *Bacillus*-derived volatiles against *Xanthomonas* species.

Numerous *B. velezensis* strains control plant pathogens via direct antagonism (Cai et al., 2017; Grady et al., 2019; Kim et al., 2022), nutrient competition (Fan et al., 2018; Rabbee et al., 2019). This efficacy stems from secondary metabolites like lipopeptides (surfactin and fengycin), synthesized by non-ribosomal peptide synthetase (NRPS) gene clusters, and the polyketide (bacillaene), which together contribute to antibacterial activity (Kim et al., 2022). In addition, they produce of surfactin and enzymes (cellulase, lysozyme, protease) that degrade fungal cell walls (Grady et al., 2019; Kim et al., 2022; Yang et al., 2023). Siderophores enhance iron competition, and acetoin reduces virulence of *Fusarium* spp. (Spantidos et al., 2025). These traits make *B. velezensis* a more environmentally friendly alternative to chemical fungicides (Köhl et al., 2019; Fira et al., 2018).

Genomic sequencing elucidates the genetic and biochemical basis underlying the beneficial traits of *B. velezensis*. Moreover, it possesses a circular genome of approximately 3.9–4.2 Mb, with a GC content approximately of 45.8–47%, encoding 3,700–4,200 proteins-coding genes approximately 12–21 biosynthetic gene clusters (BGCs) for secondary metabolites across strains (Pan et al., 2017; Choi et al., 2021; Sachin et al., 2021; Pei et al., 2023; González-Léon et al., 2024). AntiSMASH analysis of strains EB14, HNA3 revealed 12–14 BGCs for surfactin, bacillibactin, bacillaene, difficidin, and bacilysin (Sachin et al., 2021; Zaid et al., 2022). Consistently, strain FZB42 harbors 13 BGCs, supporting ISR and biofilm formation (Fan et al., 2018). Strain 9912D contains 13 BGCs for surfactin, fengycin, bacillomycin, bacillaene, and difficidin (Pan et al., 2017). In contrast, CMRP 4490 contains eight BGCs that produce similar compounds but also include macrolactin H, bacillibactin, and bacilysin (Teixeira et al., 2021). Similarly, strain VJH504 has 12 BGCs for multiple metabolites and CAZymes, aiding fungal cell wall degradation, biofilm formation and plant immunity (Yang et al., 2023). Together, these findings highlight the multifaceted genetic and biochemical capacities of *B. velezensis* as a sustainable biocontrol and plant growth-promoting bacterium, offering valuable prospects for environmentally friendly agricultural management (Fira et al., 2018; Marin et al., 2019).

Beyond strain level genomic features, pan-genomic studies have extensively characterized species within the genus *Bacillus*, with *B. velezensis* and *B. subtilis* serving as models (Kim et al., 2017; Zeng et al., 2021; Kadiri et al., 2023; Neal et al., 2024). At the species level, comparative pangenome analyses of *B. velezensis* reported core gene cluster counts of 3,566–3,592 among strains VJH504, FZB42, G341, and BvL03, with 3,295 clusters shared across all four, indicating a relatively conserved core genome (Yang et al., 2023). Furthermore, an expanded dataset of 54 *B. velezensis* strains, including VB7, identified 2,226 core genes, while the accessory genome ranged from 666 to 1,797 genes and strain-specific genes from 30 to 179, with VB7 carrying 30 unique genes, largely hypothetical or related to sporulation (Kadiri et al., 2023). In parallel, a pan-genome-scale metabolic model of 481 *B. subtilis* genomes captured strain-level variation in nutrient use and metabolic traits, allowing the classification of strains into five metabolically distinct groups (Neal et al., 2024). Finally, cross-species analysis of 238 strains from five *Bacillus* species (including *B. amyloliquefaciens*, *B. anthracis*, *B. cereus*, *B. subtilis*, and *B. thuringiensis*) highlighted consistent functional contrasts between core and strain-specific genes (Kim et al., 2017).

Previous genomic studies on *B. velezensis* have focused on individual strains, lacking synchronized comparisons among strains from the same ecosystem. This has limited insights into gene functions related to environmental adaptation. Pan-genome and functional gene network analyses remain preliminary, with core and accessory genes not yet clearly defined. AntiSMASH data have primarily identified common lipopeptide biosynthetic gene clusters (BGCs).

This study presents the first intra-species genomic comparison between two *B. velezensis* strains, BP5 and BP103, isolated from the pepper rhizosphere. Previous studies have frequently reported the antagonistic activity of this species against fungi. In the present research, both BP5 and BP103 exhibited strong antagonistic activity against *X. euvesicatoria*, the causal agent of leaf spot disease on pepper in Vietnam. Genomic analyses of the two strains revealed their genetic diversity and determinants of antagonism in *B. velezensis*, distinguished core and accessory genes, highlighted strain-specific paralogs, and identified putative gene clusters potentially associated with their antagonistic abilities.

## 2 Materials and methods

### 2.1 Isolation, pathogenicity testing, and identification of *Xanthomonas* strain

The bacterial strain was isolated from symptomatic pepper leaves showing leaf spot symptoms, collected from fields in Đống Tháp Province. Diseased tissue from the margin of necrotic lesions was streaked onto King’s B (KB) agar (HiMedia, India) and incubated at 28°C for 3–5 days. Characteristic yellow colonies typical of *Xanthomonas* spp. were selected and repeatedly streaked for purification on KB agar. Pure cultures were preserved long-term at –20°C in 30% (v/v) glycerol. The isolate was designated XP10, based on the sample code stored in the Biotechnology Laboratory at Dong Thap University.

Pathogenicity of the *Xanthomonas* sp. strain (XP10) was confirmed by infiltrating a bacterial suspension (5 × 10^8^ CFU/mL in 0.01 M MgSO_4_) into the abaxial surface of developing pepper leaves using a needleless syringe. Inoculated plants were incubated at 25°C for the first 2 days to promote infection, then maintained in a net-house. Typical leaf spot symptoms developed on infiltrated leaves. The bacterium was re-isolated from the margin of symptomatic lesions by streaking onto KB agar, confirmed by characteristic yellow colonies, thereby fulfilling Koch’s postulates.

Genomic DNA was extracted from bacterial colonies using the AllPrep DNA/RNA Mini Kit (Qiagen). The near-full-length 16S rRNA gene was amplified with primers 27F and 1492R following Weisburg et al. (1991). PCR products were sequenced on the Oxford Nanopore Technologies (ONT) platform. Raw fast5 data were basecalled to fastq format using Guppy v6.5.7 (ONT). Read quality was evaluated with NanoQ v0.10.0 (Steinig and Clarke, 2022), and low-quality reads were filtered and trimmed using Filtlong v0.2.1.

Full-length 16S rRNA consensus sequences were generated from Nanopore amplicon reads using Medaka (ONT) after basecalling with Guppy. Taxonomic assignment was performed by BLASTn alignment of the consensus sequences against the NCBI GenBank database (Camacho et al., 2009). The 16S rRNA sequence of XP10 has been deposited in GenBank with the corresponding accession number (PX836769).

### 2.2 Bacterial strains, incubation parameters, antagonistic activity assay, and scanning electron microscopy (SEM) analysis

Rhizosphere soil samples (collected at a depth of 20 cm) were obtained from twenty pepper fields in the Mekong Delta, Vietnam. Five grams of each soil sample were suspended in 50 mL of sterile saline peptone solution (0.85% NaCl) and shaken at 200 rpm for 15 min. Serial dilutions were prepared, and 50 μL aliquots from appropriate dilutions were spread onto Luria-Bertani (LB) plates (HiMedia, India). Plates were incubated at 30°C for 48 h. Well-isolated colonies were selected and purified by repeated streaking on LB. Purified isolates were preserved at –20°C in 30% (v/v) sterile glycerol, and working cultures used in all antagonism assays were freshly revived from these glycerol stocks and re-purified prior to testing.

Antibacterial activity assay against *Xanthomonas* sp. XP10 was performed using strain XP10 as the indicator bacterium. King’s B agar plates (100 mm in diameter) (King et al., 1954) were poured with approximately 15 mL of medium, which produced a uniform but not excessively thick XP10 lawn suitable for clear inhibition zone measurements. After that, bacterial suspensions of rhizobacterial isolates (OD_600_ = 0.3) were used to soak sterile filter paper disks (5 mm diameter) and placed on the disease plates, respectively. Plates were incubated at 30°C for 48 h. Sterile saline peptone solution was used as the negative control, and oxolinic acid (commercial 20% WP formulation, applied at a final concentration of 200 μg/mL) (Sang et al., 2015) served as the positive control (a locally applied bactericide commonly used for this purpose). Antibacterial activity was evaluated by measuring the diameters of inhibition zones, which were calculated by measuring the total zone diameter (including the bacterial colony) and subtracting the disk diameter (5 mm) (Thanh, 2022). The data were analyzed using MINITAB version 16.1. A one-way analysis of variance (ANOVA) was performed for each data set. Significant differences among means were determined using Tukey’s multiple comparison test at a significance level of 0.05.

Strains BP5 and BP103 were cultured on Luria-Bertani (LB) agar at 30°C for 48 h. Bacterial colonies from each strain were then harvested and separately suspended in sterile distilled saline solution (0.9% NaCl) to prepare bacterial suspensions (approximately 10^9^ CFU/mL). A 5 μL aliquot of each bacterial suspension was pipetted onto a glass coverslip using a micropipette and air-dried at 60°C for 15 min to thermally inactivate, rapidly dehydrate, and immobilize the cells on the surface. The dried coverslips were mounted onto SEM stubs using conductive carbon adhesive tape. Subsequently, the samples were sputter-coated with a thin layer of platinum (approximately 8–12 nm) at a current of 30 mA for 30 s to enhance electrical conductivity, minimize charging effects. SEM observation and imaging of overall surface morphology were performed at the Nano-Electrochemistry Laboratory, Department of Chemical Engineering, Can Tho University, using a JEOL JCM-7000 NeoScope™ benchtop scanning electron microscope (JEOL Ltd., Japan) under high-vacuum mode with secondary electron (SE) detection and standard accelerating voltage settings.

### 2.3 Biochemical traits and enzymes involved in carbohydrate metabolism

The hydrolytic enzyme activities and hydrocarbon utilization capacities of strains BP5 and BP103 were evaluated through their protease, lipase, amylase, and cellulase activities. Amylase activity was determined on starch agar (Sonar and Halami, 2014), whereas protease activity was assessed on 1% casein-supplemented agar (Rawaliya et al., 2022). Lipase activity was evaluated on LB medium containing Tween 20, Tween 80, and CaCl_2_.2H_2_O (Ramnath et al., 2017), while cellulase activity was detected using carboxymethyl-cellulose (CMC) agar (Ariffin et al., 2006).

Experiments involved applying 5-mm diameter sterile paper discs saturated with bacterial suspensions (OD_600_ ≈ 1.0) onto the respective media and incubating at 30°C for 72 h. Post incubation, casein plates were treated with 10% TCA for 10 min at room temperature to precipitate proteins and visualize protease zones; starch plates were stained with Lugol’s solution for amylase activity; CMC plates were stained with 1 mol/L Congo red for 30 min and subsequently destained with 1 mol/L NaCl for 30 min to detect cellulase activity. Extracellular enzymatic activities were assessed using specific agar plate assays, with all tests performed in triplicate to ensure reproducibility. Specifically, protease activity was indicated by the formation of clear hydrolysis zones (clear zones) around colonies on casein agar. In addition, lipase activity was detected by the appearance of white precipitate zones (or opaque halos) surrounding the colonies on lipid-containing medium. Furthermore, amylase activity was observed through transparent halos on starch agar after flooding with Lugol’s iodine solution. Moreover, cellulase activity was visualized as distinct degradation halo zones on carboxymethyl cellulose (CMC) agar following Congo red staining and destaining.

Furthermore, Siderophore production was evaluated by inoculating antagonistic strains on CAS medium (Schwyn and Neilands, 1987). Strains were incubated at 30°C for 3–5 days. Siderophore synthesis was indicated by the formation of an orange-yellow halo surrounding bacterial growth. All assays were performed in triplicate (technical replicates) using the same bacterial suspension under identical conditions.

### 2.4 Genome sequencing and annotation

*B. velezensis* strains BP5 and BP103 were grown in Luria-Bertani (LB) medium at 37°C for 24 h. Genomic DNA was isolated from the bacterial cells using the Monarch^®^ Spin gDNA Extraction Kit (NEB, United States). The DNA concentration was quantified with the QuantiFluor^®^ dsDNA System (Promega), while DNA integrity was evaluated by capillary electrophoresis on the Agilent 2100 Bioanalyzer based on fragment size distribution profiles.

Sequencing libraries were constructed using the Illumina TruSeq Nano DNA kit targeting an average insert size of 350 bp. Quality assessment of libraries was performed by analyzing PCR amplicon size distribution on the Agilent 2100 Bioanalyzer. Qualified libraries were subjected to sequencing on the Illumina NovaSeq 6000 platform (paired-end 150 bp reads) at Macrogen Corporation (Seoul, South Korea), yielding high sequencing coverage (∼733 × for BP5 and ∼577 × for BP103).

Raw paired-end sequencing generated 2.991 Gbp and 2.253 Gbp of data for strains BP5 and BP103, respectively. To ensure optimal accuracy for downstream analyses, the raw datasets underwent rigorous quality control and preprocessing using fastp (Chen S. et al., 2018). Although initial standard scanning detected no explicit adapters, the tool’s paired-end overlap analysis and poly-G trimming algorithms successfully removed adapter and artifact sequences from 17,770 reads in BP5 and 5,954 reads in BP103. Additionally, strict filtering parameters resulted in the removal of 139,656 low-quality reads, 294 N-rich reads, and 22,350 overly short reads from the BP5 dataset, while 85,626 low-quality reads, 5,500 N-rich reads, and 9,988 overly short reads were discarded from the BP103 dataset. Following this comprehensive preprocessing, the retained high-quality clean datasets comprised 2.965 Gbp for BP5 and 2.237 Gbp for BP103.

The quality of raw sequencing reads was initially checked with FastQC (v0.12.1) and then aggregated using MultiQC (v1.26). Genome assembly was carried out *de novo* using Unicycler v0.5.1 in short-read mode, which utilizes SPAdes as the underlying assembler for Illumina data. Assembly quality was then assessed by QUAST, referencing the closest available genome, *B. velezensis* 160 (GenBank: CP119675.1).

Subsequent *de novo* assembly utilizing these clean reads yielded draft genomes of 4,078,673 bp for BP5 and 3,906,643 bp for BP103, indicating that the BP103 genome is more compact, being 172,030 bp (4.22%) smaller than that of BP5.

Protein-coding sequences were predicted using the NCBI Prokaryotic Genome Annotation Pipeline (PGAP), which served as the sole reference for downstream genomic analyses. The draft genome was visualized through Proksee, a web-based tool for prokaryotic genome annotation and visualization.^1^

### 2.5 Gene function clustering

Gene functions were annotated by querying multiple protein databases. Clusters of Orthologous Genes (COG) were assigned using eggNOG-mapper (v5.0.1). Functional annotation relied on GO terms via InterPro (v5.75) and Kyoto Encyclopedia of Genes and Genomes (KEGG) pathway mapping via KAAS (Zhou et al., 2023; Xie et al., 2023). Pfam-based protein family annotation was performed with Pfam_Scan (v1.6) against Pfam (v37.1) (Finn et al., 2014).

### 2.6 Analysis of average nucleotide identity (ANI)

ANI analysis was used to assess genome-wide similarity to infer genetic relationships and species delineation at the genome level. In this study, the sequenced genomes of *Bacillus* strains BP5 and BP103 were compared with reference genomes using MUMmer v4.0.1 to quantify similarity and assess genetic diversity among strains. Given that MUMmer-based ANIm remains a widely used baseline for evaluating the accuracy of alignment-free methods, this approach provides high-resolution and reliable estimates of genome-wide nucleotide identity (Jain et al., 2018). A reference strain, *B. subtilis* DSM10 (CP120681.2), was included as an outgroup to represent a more distant relationship within *B. subtilis* species complex, along with 25 closely related *B. velezensis* genomes retrieved from GenBank/RefSeq: TZS01 (CP141828.1), SRCM123422 (CP128503.1), LOH112 (NZ_CP092110.1), 77-E7 (CP197347.1), 77-E7-BHI (CP197348.1), 77-E7-A5 (CP197349.1), PD9 (CP142142.1), 10075 (CP025939.1), XRD006 (CP118911.1), 12Y (CP120711.1), PEBA20 (CP046145.1), Lzh-a42 (CP025308.1), CIMT3 (NZ_CP169570.1), ID-A04 (CP066379.1), ID-A03 (CP066378.1), CBMB205 (NZ_CP011937.1), YJ11-1-4 (CP011347.1), FS3 (CP195723.1), FZB42 (NC_009725.2), BP1 (NZ_CP165606.1), 160 (CP119675.1), AP46 (CP160229.1), GJJK74 (CP137890.1), DSYZ (NZ_CP030150.1), and 11640 (NZ_CP026610.1). Three very closely related genomes (77-E7, 77-E7-BHI, and 77-E7-A5) from the same study were intentionally retained to capture fine-scale variation within the same lineage.

### 2.7 Phylogenetic analysis throught pangenome

Pangenome analysis of *B. velezensis* BP5 and BP103 was performed using two independent tools. PPanGGOLiN v2.2.3 was run with an identity threshold of 0.90 and a coverage threshold of 0.90, and Roary v3.13.0 was run with identically matched BLASTP parameters (90% sequence identity and 90% coverage), a core gene threshold of 99% (-cd 99), and an MCL inflation value of 1.5 (-iv 1.5), in order to define the core, shell, and cloud gene clusters. Subsequently, orthologous gene families (orthogroups) were inferred using OrthoFinder v3.1.0 across 28 Bacillus genomes, including multiple *B. velezensis* strains, *B. subtilis* DSM10, and the representative isolates BP5 and BP103. Single-copy orthologs, defined as orthogroups containing exactly one gene from each genome, were extracted and concatenated into a single amino acid alignment, which was used as input for constructing a maximum-likelihood phylogenomic tree in IQ-TREE v3.0.1. The best-fitting amino acid substitution model, LG++F+I+G4, was selected by ModelFinder. Branch support was assessed using both the SH-like approximate likelihood ratio test (SH-aLRT; 1,000 replicates) and ultrafast bootstrap (1,000 replicates). The resulting tree was visualized and annotated using iTOL v7 (Interactive Tree of Life).

### 2.8 Secondary metabolite biosynthetic gene clusters analysis

Secondary metabolite biosynthetic gene clusters in the genomes of *B. velezensis* BP5 and BP103 were predicted using antiSMASH v8.0.1. The putative products and their chemical scaffolds were inferred by homology-based comparison of the predicted clusters against entries in the MIBiG database (Wang et al., 2019; Zhou et al., 2023; Xie et al., 2023).

### 2.9 Identification of carbohydrate active enzyme (CAZymes)

Genes encoding CAZyme enzymes from families GH, PL, CE, GT, AA, and CBM in BP5 and BP103 genomes were identified with dbCAN v5.1.2 (utilizing HMMER, Hotpep, and DIAMOND) (Zhang et al., 2024). The predicted protein sequences were then queried against the Carbohydrate-Active Enzymes (CAZy) database by BLAST to assign enzyme families.

## 3 Results

### 3.1 Isolation, pathogenicity, and identification of *Xanthomonas* strain

**FIGURE 1:**
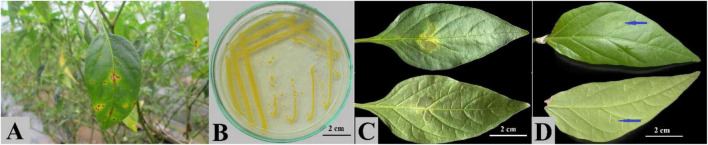
Symptoms of leaf spot caused by *Xanthomonas* sp. on pepper. **(A)** Leaf spot symptoms in the field. **(B)** Colony morphology of *Xanthomonas* sp. isolated from the diseased sample. **(C)** Pathogenicity test via leaf infiltration: adaxial (upper) and abaxial (lower) surfaces of the inoculated pepper leaf. **(D)** Negative control (infiltration with MgSO_4_ alone).

From typical symptomatic leaf samples, the isolated strain formed smooth, round, convex colonies with entire margins, buttery (mucoid) consistency, and pale yellow pigmentation (Figure 1). This mucoid, buttery appearance is characteristic of *Xanthomonas* species (Garrity, 2005; Ryan et al., 2011).

Infiltration of the bacterial suspension into pepper leaves using the needleless syringe method resulted in water-soaked lesions delimited by veins, progressing to necrosis, with mild chlorosis surrounding the necrotic areas. Microscopic examination of wet mounts from excised lesions revealed bacterial streaming, and re-isolation by streaking onto King’s B medium yielded yellow, mucoid colonies with morphology identical to the original XP10 isolate, typical of *Xanthomonas.* Taken together, these results strongly support the identification of XP10 as the causal agent of bacterial leaf spot on pepper.

The 16S rRNA gene of strain XP10 was sequenced. BLASTn comparison (NCBI, latest version) of the pepper strain sequence against GenBank showed the closest match to *X. euvesicatoria* DSM 19128 (accession NR_114773), with 99.93% identity. High-identity matches were also observed with other *Xanthomonas* spp. (e.g., *X. perforans*, *X. citri*, *X. alfalfae*, *X. vasicola*), confirming placement within the genus *Xanthomonas*. It has been identified as the *X. euvesicatoria* strain. The 16S rRNA sequence data for XP10 have been deposited in GenBank under accession number PX836769 (NCBI).^2^

### 3.2 Antagonistic and morphological features of the BP5 and BP103 strains

**FIGURE 2:**
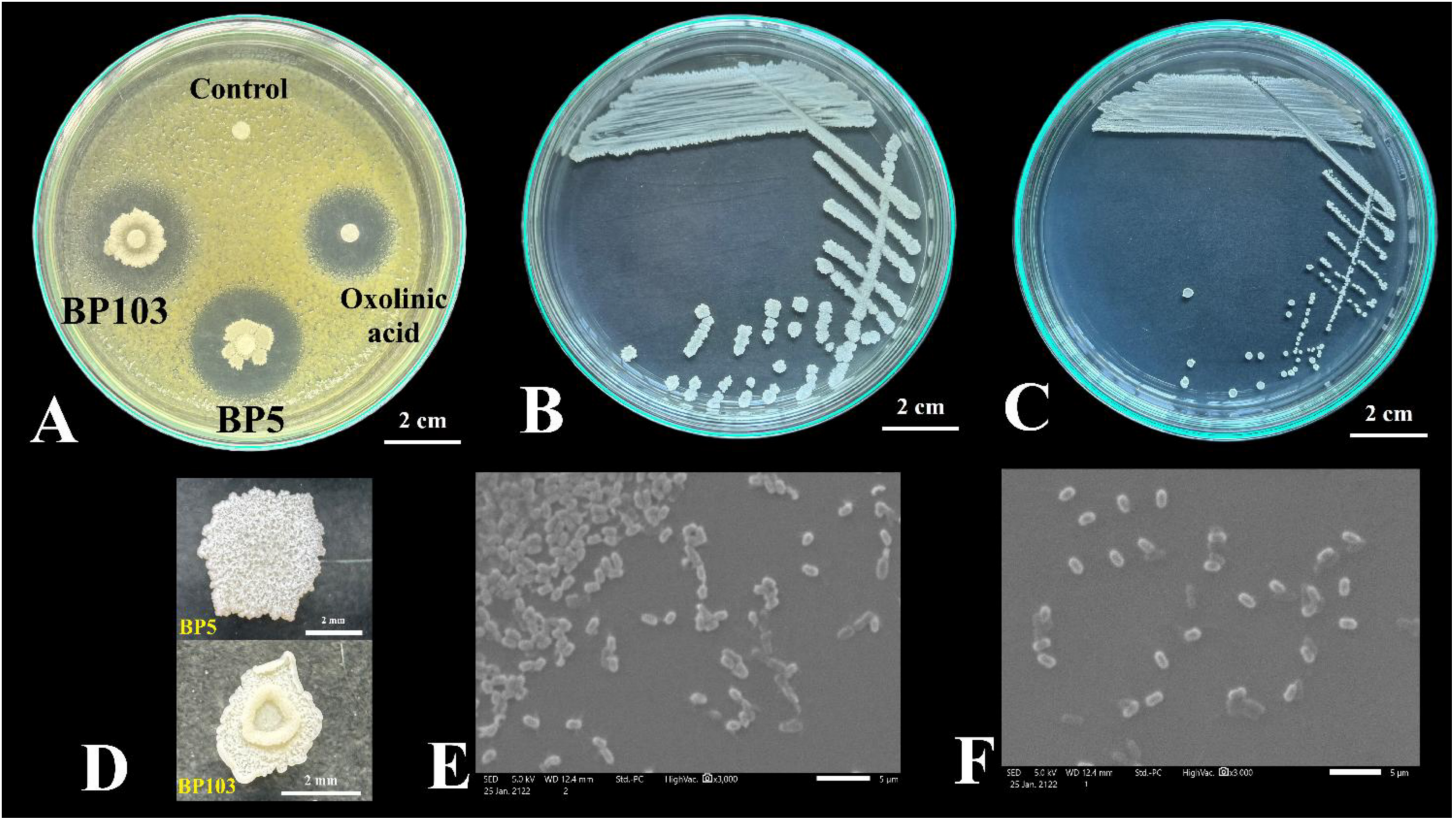
Antagonistic and morphological characteristics of *B. velezensis* BP5 and BP103. **(A)** Inhibition zones indicating growth suppression of BP5 and BP103 compared with oxolinic acid. **(B**,**C)** Colony morphology of BP5 and BP103, respectively. **(D)** Close-up of a bacterial colony: BP5 (above), BP103 (below). **(E)** Scanning electron microscopy (SEM) images of BP5 (scale bar = 5 μm; magnification = 3,000×). **(F)** SEM images of BP103 (scale bar = 5 μm; magnification = 3,000×).

A dual-culture antagonism assay was performed with 283 bacterial isolates collected from rhizosphere soil to evaluate their activity against *Xanthomonas* sp. XP10, the causal agent of bacterial leaf spot on pepper. Among these 283 isolates, 25 (8.8%) showed potential antagonistic effects, and 12 of them exhibited inhibition zones over 20 mm in diameter, with BP5 and BP103 showing the strongest inhibition of pathogen growth (Figure 2 and Table 1). Notably, BP5 produced the largest inhibition zone (36.7 mm), which was significantly greater than that of the positive control, oxolinic acid (31.3 mm). In contrast, BP103 (32.1 mm) showed a comparable level of inhibition to oxolinic acid, with no statistically significant difference observed between them. Both strains are Gram-positive, short rod-shaped bacteria (Figure 2) that form milky white, circular, and irregular colonies on LB agar plates. The other antagonistic isolates were not further characterized at the species level within the scope of this study, and thus our subsequent analyses focused on BP5 and BP103.

**TABLE 1:** Diameters of inhibition zones (mm) against *Xanthomonas* sp. XP10 by 25 rhizobacterial isolates with antagonistic activity after 48 h of incubation.

Antagonistic bacteria	Diameter of the inhibition zone (mm)
BP5	36.7^a^
BP11	24^de^
BP18	23^def^
BP27	25.7^cd^
BP65	22.7^def^
BP78	20^ef^
BP103	32.1^b^
BP111	26^cd^
BP135	20^ef^
BP207	26,3^cd^
BP234	26.3^cd^
BP277	26.1^cd^
Positive Control	31.3^b^

Means in the same column followed by the same letter are not significantly different according to Tukey’s test (*P* ≤ 0.05) using Minitab (v16.1). Each value represents the mean of three replicates. Positive control: Filter paper disks were impregnated with 20% oxolinic acid solution instead of bacterial suspensions.

### 3.3 Determination of the biochemical activity of the BP5 and BP103 strains

**FIGURE 3:**
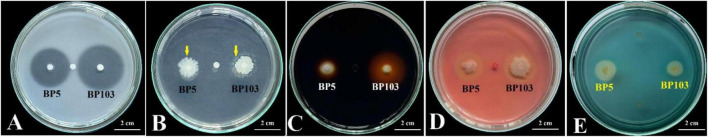
Detection of antagonistic enzymes and secondary metabolites in strains BP5 and BP103. **(A)** Protease activity with clear zones on casein agar. **(B)** Lipase activity with white precipitates around colonies. **(C)** Amylase production with transparent halos after Lugol’s treatment. **(D)** Cellulase activity with degradation halo zone after congo red staining. **(E)** Siderophore production with yellow halos on CAS medium.

The biochemical characteristics of strains BP5 and BP103 were evaluated through *in vitro* assays. Both strains demonstrated the ability to produce several extracellular enzymes, including protease, lipase, amylase, and cellulase, as evidenced by the formation of hydrolysis zones, white precipitates, and degradation halos on the respective media (Figures 3A–D). The formation of yellow halos on CAS medium (Figure 3E) indicated the production of siderophores by these strains, based on the qualitative CAS assay. These results are based on qualitative observations from plate assays, and no quantitative measurements or statistical analyses were performed. Therefore, no comparative assessment between the two strains was made. Overall, the findings suggest that both BP5 and BP103 possess metabolic capabilities related to the production of extracellular enzymes and bioactive compounds. Furthermore, future work could investigate how these metabolites contribute to survival and biocontrol efficacy through complementary *in vitro* assays.

### 3.4 Genomic features of BP5 and BP103

Whole-genome sequencing of *Bacillus velezensis* strains BP5 and BP103 generated raw data of 2.991 Gbp (Q30 = 92.69%) and 2.253 Gbp (Q30 = 92.37%), respectively. After quality filtering, the clean data reached 2.964 Gbp (Q30 = 93.00%) for BP5 and 2.237 Gbp (Q30 = 92.48%) for BP103. *De novo* assembly using QUAST yielded high-quality draft genomes, with BP5 having a total length of 4,078,673 bp, N50 of 564,984 bp, 31 contigs, and GC content of 46.04%, while BP103 achieved a total length of 3,906,643 bp, N50 of 1,000,639 bp, 16 contigs, and GC content of 46.46%. The 172 kb (4.2%) difference in genome size between BP5 and BP103 is consistent with the high genomic plasticity of *B. velezensis* (typically 3.9–4.2 Mb), which is largely driven by the accessory genome. Our data reveal that BP5 possesses more mobile elements (5 prophages vs. 2 in BP103) and strain-specific genes (456 vs. 203 shell gene families), accounting for most of this extra genomic content. Additionally, since these are Illumina-based draft genomes, unresolved assembly gaps in highly repetitive regions may also marginally contribute to this size variation. Furthermore, based on the high-quality clean data utilized for the *de novo* assembly, the estimated sequencing coverage was exceptionally high, reaching approximately 727 × for BP5 and 573 × for BP103 (2.965 Gbp/4.078 Mbp and 2.237 Gbp/3.906 Mbp, respectively). This deep coverage effectively ensures the reliability, completeness, and high accuracy of the resulting draft genomes, thereby providing a robust foundation for subsequent downstream genomic analyses, including comparative studies.

**FIGURE 4:**
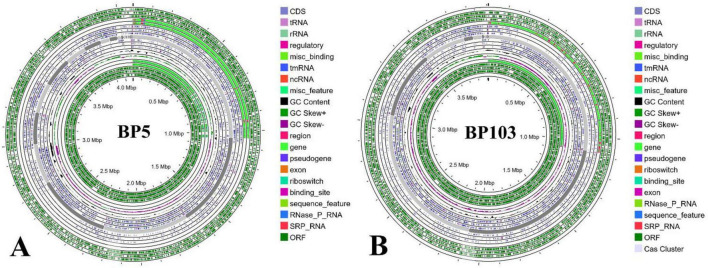
Circular genome maps of *Bacillus velezensis* strains BP5 **(A)** and BP103 **(B)**. The outer rings show the distribution of predicted coding sequences (CDSs) on the forward and reverse strands, while RNA genes (tRNA, rRNA, tmRNA, and ncRNA) and other annotated features are indicated by different colors as shown in the legend. The genome scale (Mbp) is displayed around the map. Genome maps were generated using the Proksee.

The key genomic features of *B. velezensis* strains BP5 and BP103 are outlined in Figure 4 and Table 2. Both strains share identical ncRNA (4) and rRNA (3) counts, with similar tRNA numbers (BP5: 74; BP103: 76), reflecting conserved gene regulation and translation mechanisms. It should be emphasized that the annotation of only 3 rRNA sequences in both BP5 and BP103 primarily reflects technical limitations associated with Illumina short-read data rather than a biological anomaly. The closely related reference strain *B. velezensis* 160 (GenBank: CP119675.1), although reported as a complete continuous genome and sequenced using Illumina technology (González-Léon et al., 2024), is likewise annotated with only 5 rRNA sequences. To accurately determine the copy number and structural organization of these repetitive rRNA operons, future updates of these genome records will require long-read sequencing data. BP5, with a larger genome (4,223 total genes, 4,047 protein-coding genes) and 5 prophage elements, contrasts with BP103 (3,877 total genes, 3,793 protein-coding genes, 2 prophage), despite fewer pseudogenes (64 compared with 66), indicating potential genomic and evolutionary divergence, as well as differences in the conservation of horizontally acquired genes between the two strains. Furthermore, it is important to acknowledge that predicting prophage regions (5 in BP5 and 2 in BP103) is inherently constrained by the fragmentation typical of short-read assemblies. To mitigate this technical limitation and maximize prediction accuracy, we cross-validated our findings using multiple robust prophage identification tools, specifically the PHASTER web server and the locally executed PhiSpy algorithm (Akhter et al., 2012). PhiSpy enhances detection by combining similarity- and composition-based search strategies. Despite employing these rigorous cross-checks, unresolvable repetitive sequences in Illumina data mean these regions may still be fragmented. Therefore, the reported counts should be strictly interpreted as predicted prophage-like regions rather than completely resolved intact viral genomes. As with the rRNA operons, future incorporation of long-read sequencing technologies will be necessary to accurately reconstruct the complete architecture of these mobile genetic elements.

**TABLE 2:** Genomic comparative features among *B. velezenis* BP5, BP103, 160, and *B. subtilis* DSM10.

Genome characteristics	*B.velezensis* BP5	*B.velezensis* BP103	*B.velezensis* 160	*B.subtilis* DSM10
Accession number	CDRNYM010000000 (ENA)	CDRNYL010000000 (ENA)	CP119675.1 (NCBI)	CP120681.2 (NCBI)
Size (base pairs)	4,078,673	3,906,643	4,296,610	4,295,479
DNA G+C (%)	46.04%	46.46%	45.85%	43.34%
Total genes	4223	3877	4337	4534
Protein encoding genes	4047	3793	4239	4411
Prophage	5	2	11	4
Pseudo genes	64	66	71	124
rRNA	3	3	5	30
ncRNA	4	4	4	4
tRNA	74	76	88	88

The whole genomic data obtained in the Data availability statement.

Compared to *B. velezensis* 160, BP5 and BP103 exhibit smaller genomes, higher G+C content, and reduced counts of genes, prophages, pseudogenes, rRNA, and tRNA, indicating a more constrained functional capacity. Notably, all four strains (BP5, BP103, 160, and *B. subtilis* DSM10) share 4 ncRNA genes, highlighting conserved regulatory mechanisms, yet BP5 and BP103 have fewer tRNA genes (74 and 76) than DSM10 and 160 (both 88), alongside smaller genomes and lower gene counts, implying a lower degree of functional diversity and evolutionary differentiation.

### 3.5 *Bacillus velezensis* BP5 and BP103: identification via phylogenetic and ANI analysis

**FIGURE 5:**
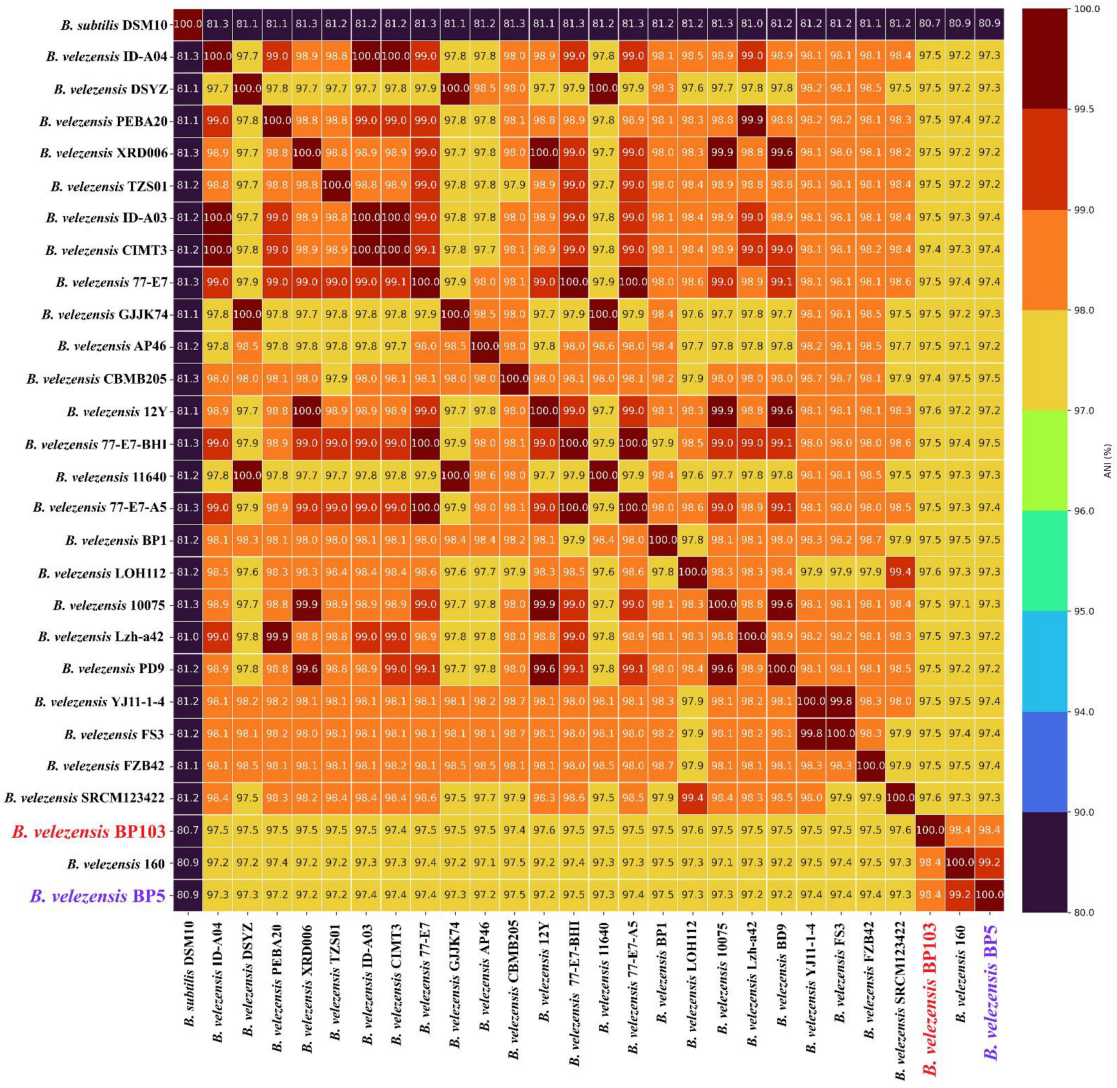
Heatmap of average nucleotide identity (ANI) values for whole-genome comparisons of antagonistic strains BP5 and BP103, *B. subtilis* DSM10 (outgroup), 25 closely related *B. velezensis* strains.

The phylogenetic tree constructed from whole-genome sequences of *B. velezensis* strains, with *B. subtilis* DSM10 (CP120681.2) as an outgroup, revealed a close evolutionary relationship among BP5, BP103, and *B. velezensis* 160 (Supplementary Figure S1). Average nucleotide identity (ANI) values (Figure 5) ranged from 84.1% relative to the outgroup *B. subtilis* DSM10 to 99.4% among the *B. velezensis* strains, indicating high genetic similarity within this species. The genomes of BP5 and BP103 showed 98.6% ANI to each other, while BP5 and BP103 exhibited ANI values of 99.4 and 98.6%, respectively, to B. velezensis 160. These high ANI values are consistent with their classification as *B. velezensis*, and only minor differences in relatedness to strain 160 were observed.

### 3.6 Gene function and protein domain annotation

Genomic functional analysis revealed a very high degree of similarity between BP5 and BP103. In the COG classification (Supplementary Figures S2A,B), the largest category corresponded to functions unknown (941 families in BP5 and 931 in BP103), followed by transcription and amino acid transport and metabolism. BP5 contained a slightly higher number of COG families related to DNA replication, recombination, and repair (approximately 20 more families), secondary metabolite biosynthesis, transport, and catabolism (about 10 more families), and cell wall/membrane/envelope biogenesis (about 9 more families) than BP103. GO (Supplementary Figures S2C,D) primarily distributes between BP5 and BP103 in biological process (765 families in BP5 and 755 families in BP103), molecular function (738 vs. 736 families), and cellular component (655 vs. 653 families), with metabolic process, catalytic activity, and cellular process prominent; BP5 slightly leads in biological/cellular process (more 6–10 families). KEGG (Supplementary Figures S2E,F) shows similarity in metabolic pathways, biosynthesis of secondary metabolites/antibiotics, and microbial metabolism in diverse environments. Comparison with reference strains BP5 and BP103 have fewer genes than YC89 but excel in antibiotic biosynthesis, secondary metabolites, and microbial metabolism functions, while Lzh-5 is rich in carbohydrate, amino acid, cofactor, and vitamin metabolism, and membrane transport. In future work, statistical tests could be applied to assess the significance of these differences.

Within the secondary metabolite biosynthesis/transport/catabolism category from COG analysis, which is more abundant in BP5 than in BP103 (Supplementary Table S1), BP5 harbored several gene families associated with secondary metabolite biosynthesis. These included genes annotated as srfAA and srfAB, encoding subunits of surfactin synthase belonging to a nonribosomal peptide synthetase (NRPS) system and involved in the biosynthesis of the lipopeptide surfactin; two genes encoding type I polyketide synthases that catalyze polyketide biosynthesis, a diverse group of secondary metabolites; and a gene annotated as *yoaI*, assigned to COG2368, which is predicted to encode an aromatic ring hydroxylase/dioxygenase based on sequence homology and COG classification. The specific role of this gene in BP5 will need to be further confirmed experimentally in future studies.

**FIGURE 6:**
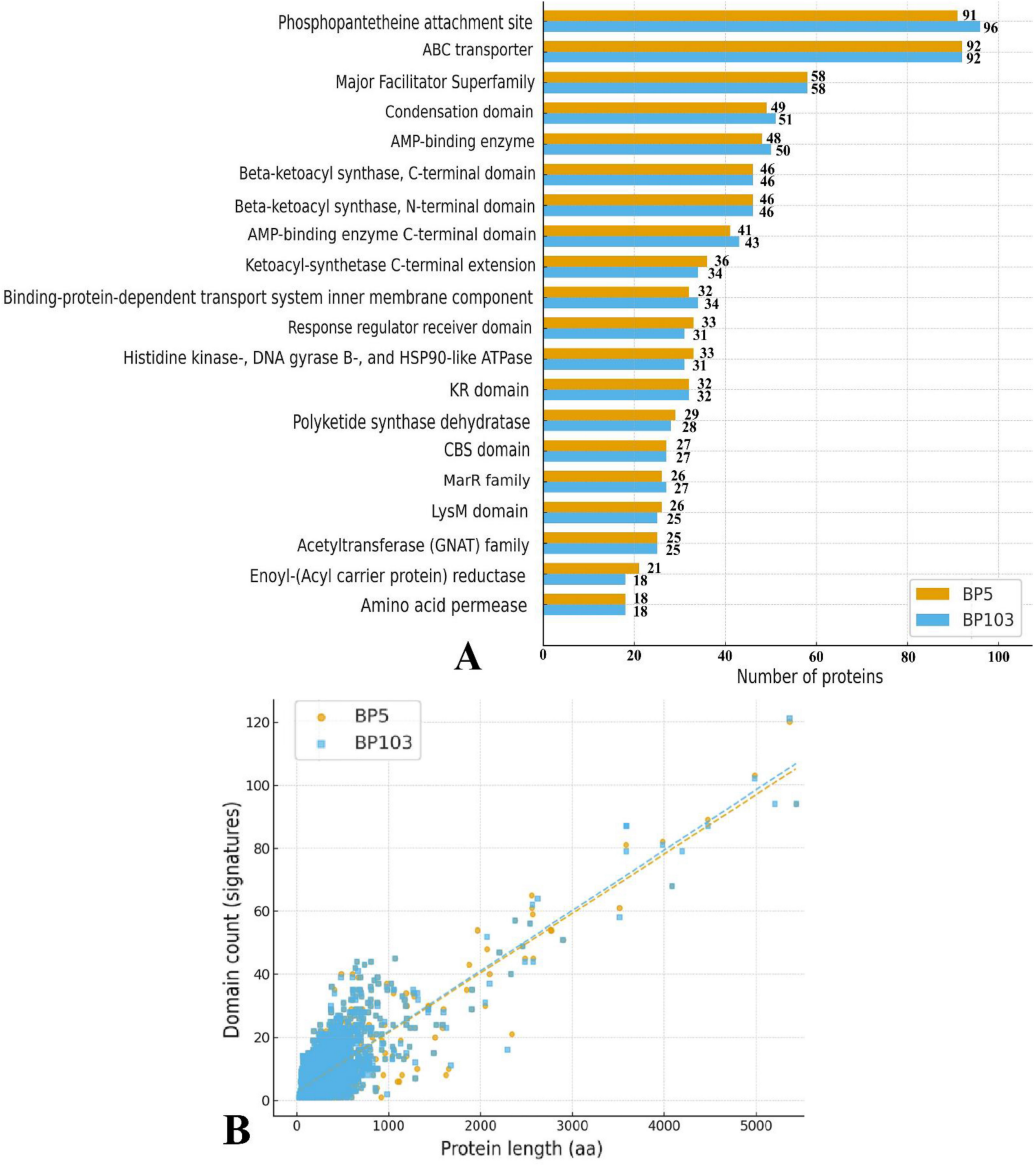
Protein domain characteristics between *B. velezensis* BP5 and BP103. **(A)** Comparison of Top 20 Pfam Domains between *B. velezensis* BP5 and BP103. **(B)** Scatter Plot Comparing Protein Length and Domain Count in BP5 and BP103.

Protein counts for the 20 most common Pfam domains in BP5 and BP103 (Figure 6A) are highly conserved, with most Pfams differing by fewer than two proteins between strains. Only the “Phosphopantetheine attachment site” domain showed a small difference, with BP103 (96 proteins) exceeding BP5 (91 proteins). This domain is typically associated with NRPS and PKS enzymes; however, the functional relevance of this minor difference remains unclear and needs to be further verified by additional analyses, such as biosynthetic gene cluster (BGC) analysis.

The relationship between protein length and domain count in BP5 and BP103 (Figure 6B) follows a linear trend: longer proteins tend to contain more domains. The data distribution is quite consistent, as indicated by nearly overlapping regression lines, reflecting a high similarity in domain structure between the two strains. Outlier proteins in BP103 suggest that this strain has greater potential for enzyme functional diversity, especially in complex secondary biosynthetic processes. The comparison of large proteins ( > 2000 aa) between BP5 and BP103 reveals only a few notable differences, mainly involving NRPSs and domain-rich proteins (Supplementary Table S3). Bacillomycin-like NRPS proteins are generally conserved in length and domain architecture, with BamB-like NRPS/PKS proteins being almost identical between BP5 and BP103, whereas a single NRPS protein with similarity to BamC at the protein/domain level was detected only in BP103. However, the presence of a complete bacillomycin biosynthetic gene cluster still needs to be investigated further. In addition, BP103 harbors an extra large NRPS (3591 aa) and an NTTRR-F1 domain-containing protein (approximately 2294 aa) that are not detected in BP5, suggesting BP103-specific large proteins. Conversely, BP5 possesses a large, domain-rich protein (approximately 2343 aa) that is absent from BP103, indicating a BP5-specific large protein. These subtle, strain-specific differences may underlie fine-scale variation in secondary metabolite repertoires or environmental adaptation of BP5 and BP103, but functional confirmation will require more detailed domain-level analyses.

### 3.7 Genetic diversity analysis based on ortholog genes and pangenome

**FIGURE 7:**
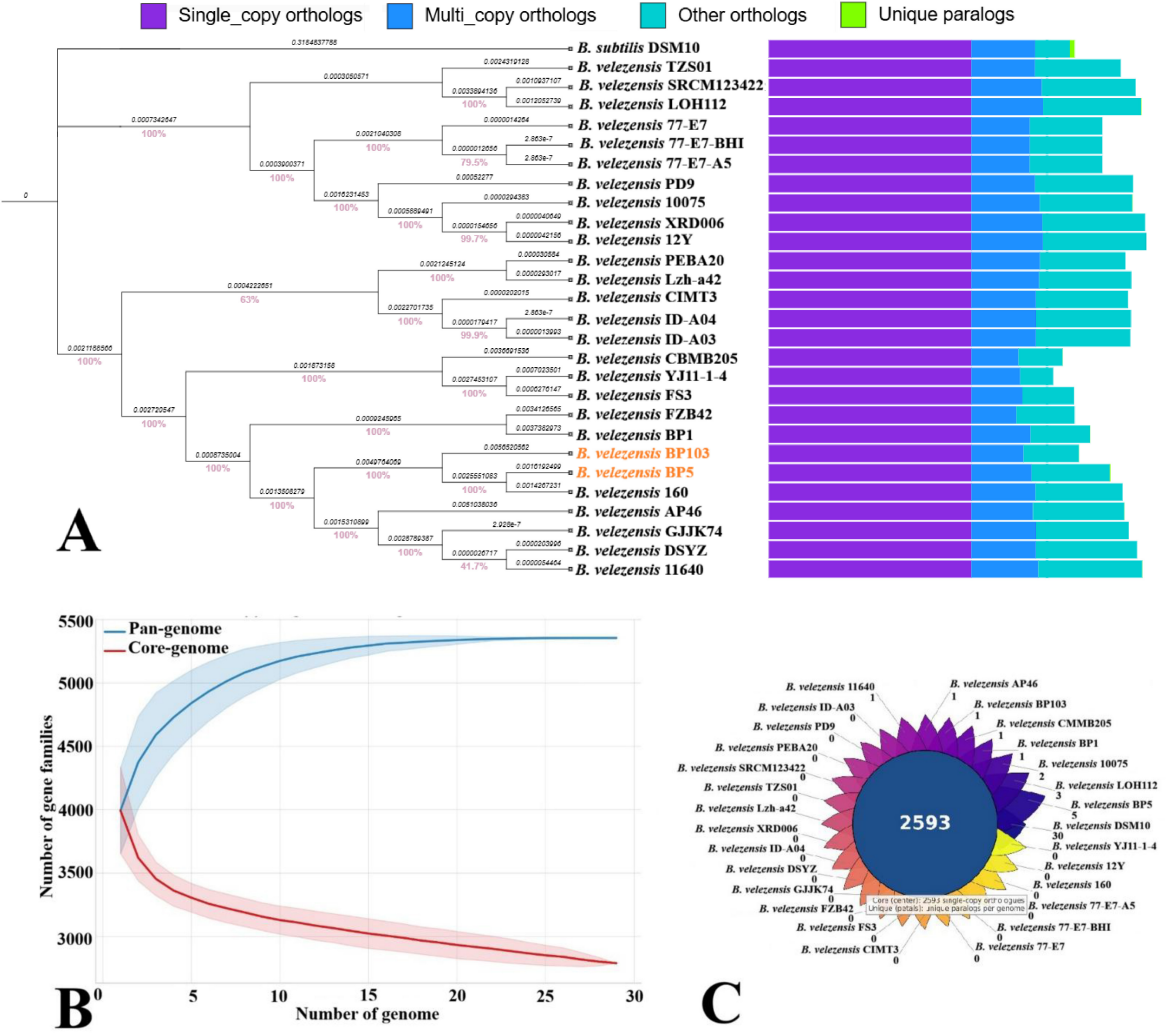
Orthology-based genetic diversity and pan-genome structure of *B. velezensis* strains BP5 and BP103. **(A)** Orthogroup classification of genomes from *B. velezensis* BP5, BP103 and related strains. **(B)** Analysis of pan-genome and core-genome of 28 *Bacillus* strains. **(C)** Consistent with the flower plot, BP5 contained five unique paralogous orthogroups (10 CDS total; two copies per orthogroup), whereas BP103 contained one unique paralogous orthogroup (2 CDS total). Functional annotation of these unique paralogs showed that BP5-specific orthogroups include a phage-associated tail tape measure protein (OG0005197; two copies), a cell-wall remodeling enzyme annotated as a transglycosylase carrying an SLT domain together with a hypothetical protein harboring a DUF/hydrolase-like region (OG0005203; one copy each), and proteins with predicted secondary-metabolism–related domains, including a ketoreductase (OG0005209; two copies) and condensation domain-containing proteins (OG0005210; two copies), as well as an additional hypothetical protein orthogroup (OG0005211; two copies). In contrast, BP103 harbored a single unique paralogous orthogroup annotated as condensation domain-containing proteins (OG0005183; two copies), but a direct functional link between these phage-related paralogs and prophage content has not yet been demonstrated.

BP5 and BP103 each possess 2,593 single-copy orthologs, comparable to other *B. velezensis* strains in the dataset, reflecting a high level of species-level genetic conservation. In the phylogenomic tree (Figure 7A), BP5 and BP103 are part of a closely related sub-cluster within the *B. velezensis* clade; the internal branch lengths separating BP5, BP103, and strain 160 range from approximately 4 × 10^−3^ to 6.7 × 10^−3^ substitutions per site, indicating a much closer evolutionary relationship among BP5, BP103, and 160 than between this group and the other *B. velezensis* genomes (Figure 7A). However, BP103 has fewer multi-copy orthologs (336) and other orthologs (763) compared to BP5 (388 and 910, respectively) and most other strains (Supplementary Table S3). In contrast, BP5 has a higher number of unique paralogs (5) than BP103 (1), suggesting that BP5 has acquired a small set of strain-specific genes, although these still represent only a minor fraction of the genome (Figure 7C). The pan-genome analysis of BP5, BP103, *B. subtilis* DSM10 and 25 additional *B. velezensis* strains showed a decreasing core-genome curve and an increasing pan-genome curve as more genomes were added (Figure 7B). The core-genome size gradually decreased while the pan-genome size increased as more genomic data were added. This reflects the increasing number of gene families with each addition of *B. velezensis* genomic data to the analysis. Overall, BP5 and BP103 maintain a closely related orthogroup structure, but BP5 exhibits a more pronounced trend toward genetic divergence.

**FIGURE 8:**
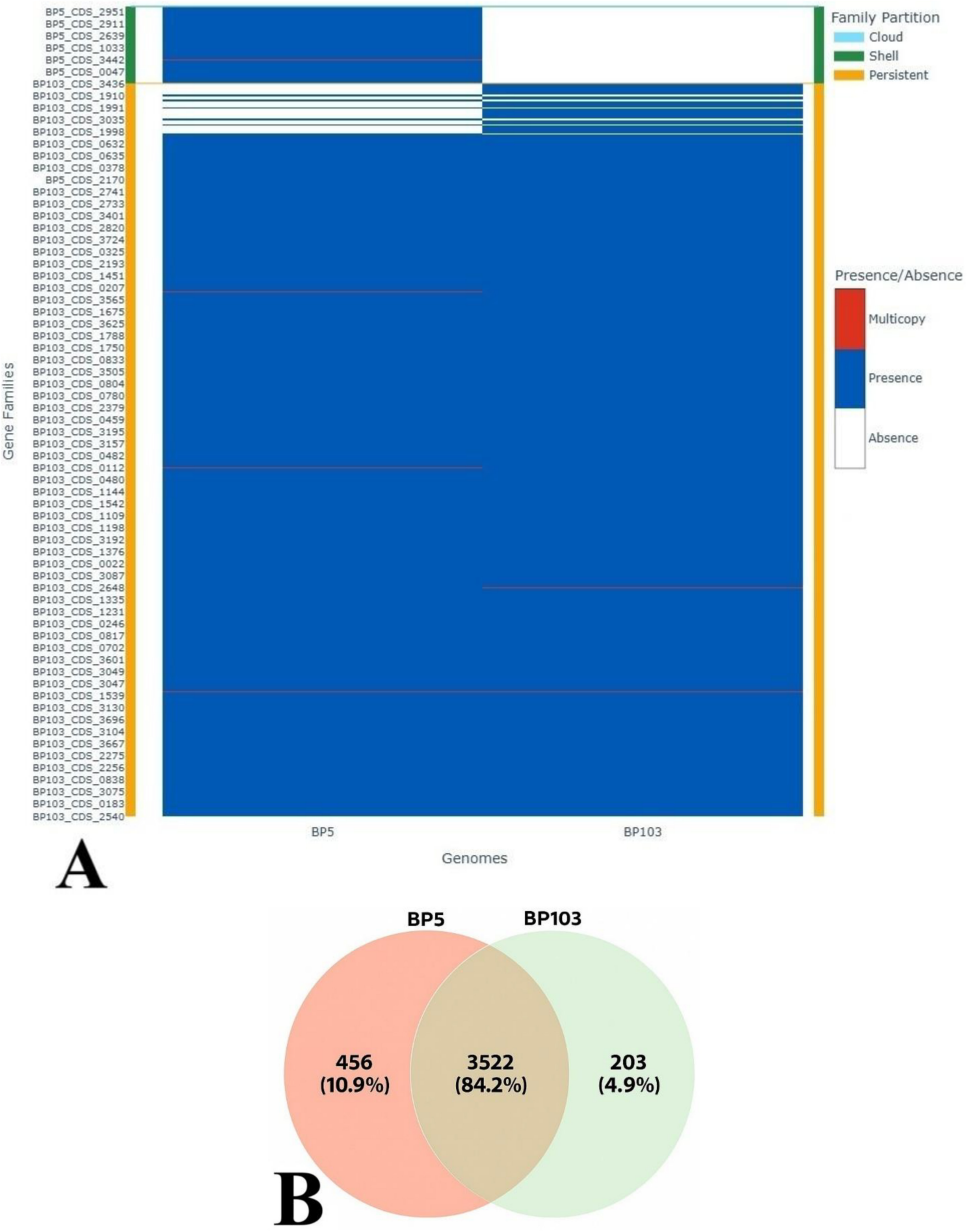
Distribution and comparison of gene families in *B. velezensis* strains BP5 and BP103. **(A)** Heatmap of orthogroup in *B. velezensis* BP5 and BP103 genomes, with gene family and strain-specific annotations. **(B)** The Venn diagram depicts the core and unique gene families between *B. velezensis* BP5 and BP103.

The distribution of gene families across the genomes of *B. velezensis* BP5 and BP103 and related *B. velezensis* strains (Supplementary Figure S3) reveals that most genes belong to the persistent group (core genes), indicating a high degree of conservation between the two microbial strains. Gene families unique to each genome are represented, corresponding to cloud or shell genes (Figure 8A). Specifically, BP5 contains 456 shell gene families absent in BP103, whereas BP103 has 203 shell gene families (Figure 8B). Overall, while BP5 and BP103 share a highly conserved orthogroup structure, BP5 shows a slightly greater tendency toward genetic divergence driven by unique paralogs and cloud genes.

Genome analysis of strains BP5 and BP103 reveals that they share 3,593 core gene families, indicating high conservation of essential functions and strong genetic similarity (Table 3), and this value is comparable in magnitude to previously reported core genome sizes for *B. velezensis*, suggesting that BP5 and BP103 are representative members of the species complex, although no formal statistical comparison was performed. Among these, the hypothetical/unknown (844 genes, 23.96%) and Other (792 genes, 22.49%) groups account for nearly 50%, highlighting that many fundamental genes remain insufficiently annotated. Although the “hypothetical/unknown” fraction appears large, it mainly reflects the presence of widely conserved hypothetical proteins in the *B. velezensis* pan-genome, many of which are also annotated as hypothetical in reference genomes and curated databases. In addition, the “Miscellaneous functional classes” (Other) category aggregates diverse low-frequency functional classes that do not fit into the major groups defined in our manual classification scheme. Together, these categories indicate that a substantial proportion of core genes in *B. velezensis* remain incompletely characterized at the functional level. Genes related to energy/central metabolism (408 genes, 11.58%), Protein secretion/export (359 genes, 10.19%), transcriptional regulation (215 genes, 6.10%), DNA replication/repair (121 genes, 3.44%), and transporters (81 genes, 3.30%) are highly represented, supporting the core functions of *B. velezensis*. Additionally, cell wall proteins (50 genes, 1.42%), stress response/chaperones (37 genes, 1.05%), and anti-phage defense (9 genes, 0.26%) contribute to basic adaptive capabilities. Notably, the sporulation group (352 genes, 9.99%) and secondary metabolites (NRPS/PKS, 55 genes, 1.56%) enhance survival and biocontrol potential in *B. velezensis*.

**TABLE 3:** Distribution of functional groups in the core genome.

Functional group	Number of core families	Percentage in core (%)
Hypothetical/unknown	844	23.96
Miscellaneous functional classes	792	22.49
Energy and central metabolism	408	11.58
Protein secretion/export	359	10.19
Sporulation	352	9.99
Transcription/Regulation	215	6.10
Translation/Ribosome	149	4.23
DNA replication/repair	121	3.44
Transporters	81	2.30
Secondary metabolites (NRPS/PKS)	55	1.56
Cell wall/envelope	50	1.42
Membrane proteins	37	1.05
Stress response/chaperones	30	0.85
Cell division	20	0.57
Defense (anti-phage)	9	0.26

Analysis of the distribution of functional genes within the unique gene sets of strains BP5 and BP103 (Supplementary Tables S4, S5) revealed a similar overall pattern, which was dominated by genes encoding hypothetical or uncharacterized proteins. This category accounted for 365 genes in BP5 and 132 genes in BP103. Meanwhile, the remaining unique genes of both strains were assigned to several fundamental functional groups. Specifically, BP5 contained higher numbers of genes involved in genetic information processing (28 vs. 13 genes in BP103), metabolism/secondary metabolites (23 vs. 15 genes), and mobile genetic elements/phages (5 vs. 1 gene). In contrast, BP103 possessed more genes related to signal transduction/defense mechanisms (22 vs. 18 genes in BP5) and cellular processes/transport (20 vs. 17 genes). Although BP5 harbors a larger number of unique genes (456 vs. 203 in BP103), the two strains still exhibit distinct functional characteristics. While our analysis of the functional categories of these unique genes is based only on manual functional classification, the observed differences are purely descriptive and will require further validation through a formal functional enrichment test.

Consistent with these functional assignments, the genes involved in secondary metabolite biosynthesis in both BP5 and BP103 indicate that these strains possess substantial potential for producing antibacterial peptides. In BP5, the presence of BP5_CDS_2286 (tyrocidine synthase 3) together with BP5_CDS_2287 (linear gramicidin synthase subunit D) suggests the capacity to generate both cyclic and linear peptides with strong antibiotic activity. Notably, tyrocidine and gramicidin are primarily known from *Brevibacillus brevis*, and the presence of homologous genes in *B. velezensis* BP5 may reflect acquisition through horizontal gene transfer, which will require further functional validation in future studies. By contrast, BP103 carries biosynthetic genes associated with the lantibiotic mersacidin and enzymes involved in its post-translational modification and maturation. In addition, genes related to the synthesis of linear gramicidin-like peptides were also detected, together with a gene currently annotated as an indolmycin-related methyltransferase homolog. The presence of these homologous genes and the putative genetic basis for the synthesis of mersacidin, gramicidin, and indolmycin will require confirmation by future comparative genomics and functional/metabolomic analyses.

### 3.8 Prediction of secondary metabolite gene clusters in *B. velezensis* BP5 and BP103

The biosynthetic gene clusters of *B. velezensis* BP5 and BP103 were identified using antiSMASH. Both BP5 and BP103 carry 13 secondary metabolite biosynthetic gene clusters with conserved genetic organization (Table 4 and Supplementary Table S6). In line with this species-wide pattern, BP5 and BP103 each harbor 13 BGCs, positioning them toward the lower end of the reported BGC range for *B. velezensis*. Based on these predictions, the two strains display broad biosynthetic potential for characteristic *Bacillus* antibiotics, including surfactin, fengycin, bacillaene, macrolactin, difficidin, bacilysin, mersacidin, bacillibactin, and locillomycin. BP5 and BP103 also contain four distinct terpene and PKS-type gene clusters (clusters 4, 5, 9, and 11) that show no significant similarity to known BGCs. Consistent with this, genome mining using antiSMASH did not reveal a canonical, complete bacillomycin biosynthetic gene cluster in either BP5 or BP103. Overall, the presence of these predicted BGCs indicates substantial biosynthetic potential, which will require experimental validation of the corresponding metabolite production through LC-MS analyses and bioassay-guided fractionation.

**TABLE 4:** Comparative analysis of secondary metabolite gene clusters among *B. velezensis* BP5, BP103.

Gene cluster	Type	Size (nt)	Most similar cluster	Bioactivity	Presence (+) or absence (−)	
					BP103	BP5
1	NRPS + betalactone	86,792	Fengycin	Antibacterial and antifungal	+	+
2	TransAT-PKS + NRPS + NRPS-like + T3PKS	110,124	Bacillaene	Antibacterial and antifungal	+	+
3	TransAT-PKS	88,201	Macrolactin H	Antibacterial	+	+
4	Terpene	20,740	–^a^	–^a^	+	+
5	PKS-like	41,245	–	–	+	+
6	Terpene-precursor + NRP-metallophore + NRPS + RiPP	65,361	Bacillibactin	Ron-acquisition and antibacterial	+	+
7	Other	41,419	Bacilysin	Antibacterial	+	+
8	Lanthipeptide-class-II	23,189	Mersacidin	Antibacterial	+	+
9	T3PKS	41,101	–	–	+	+
10	TransAT-PKS	106,167	Difficidin	Antibacterial	+	+
11	Terpene-precursor	20,891	–	–	+	+
12	NRPS	65,411	Surfactin	Antibacterial	+	+
13	NRPS + transAT-PKS	77,727	Locillomycin (B/C)	Antifungal	+	+

The “Type” column was standardized using the “A + B” notation for hybrid/multi-class BGCs, and genomic coordinates together with homology metrics (antiSMASH/MIBiG similarity, %) were added for the four previously unassigned terpene/PKS-related clusters (clusters 4, 5, 9, and 11).

^a^No matching items.

### 3.9 Characterization of the CAZyme repertoire in the genomes of *Bacillus velezensis* BP5 and BP103

**FIGURE 9:**
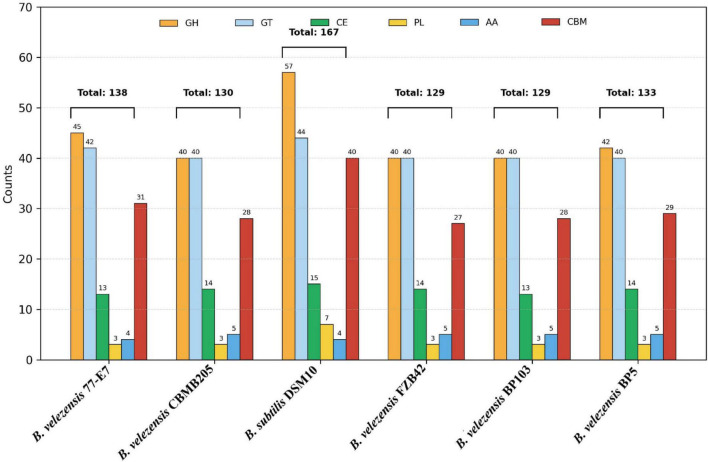
CAZyme family composition in the genomes of *B. velezensis* BP5, BP103, and related strains. The six major enzyme families are displayed with different colors with GH (glycoside hydrolases), GT (glycosyltransferases), CE (carbohydrate esterases), PL (polysaccharide lyases), AA (auxiliary activities), CBM (carbohydrate-binding modules).

Among the six *Bacillus* strains examined, *B. velezensis* BP5 possesses 133 CAZyme genes, slightly more than the commercial strain FZB42 (129 genes), while BP103 has 129 genes, equivalent to FZB42 (Supplementary Table S7 and Figure 9). BP5 exceeds BP103 by exactly four genes due to higher numbers of GH (2), CBM (1), and CE (1), mainly reflecting additional assignments to GH23, CE12, and CBM50; meanwhile, the GT, PL, and AA families are identical across all three strains. The number of CBMs is also noteworthy, with BP5 (29) and BP103 (28) both exceeding the commercial strain FZB42 (27 CBMs). Overall, BP5 and BP103 display CAZyme profiles that are broadly comparable to FZB42. The enzymatic potential of these genes will need to be verified through enzyme activity assays and in planta performance tests.

## 4 Discussion

In recent years, biological control has become a major research focus in sustainable agriculture due to its safety, environmental friendliness, and potential to replace chemical pesticides (Yang et al., 2023). Among biological control agents, *B. velezensis* is widely recognized for its high genetic stability, broad environmental distribution, and important roles in promoting plant growth and suppressing pathogens (Ye et al., 2018). Furthermore, this species is capable of synthesizing diverse array of bioactive compounds, including lipopeptides, polyketones, antimicrobial peptides, and volatile compounds, which contribute to increased crop yields and reduction of diseases caused by microorganisms. In addition, the pathogen suppression mechanisms of *B. velezensis* are further supported by extracellular enzymes such as protease, cellulase, and amylase, which can degrade and disrupt the cell wall structure of pathogens. Finally, genome analysis of novel strains provides genetic insights into these traits (Shen et al., 2013).

In the present study, two *B. velezensis* strains, BP5 and BP103, isolated from the rhizosphere of healthy pepper plants, exhibited strong antagonism against *Xanthomonas* sp., the primary causal agent of bacterial leaf spot on pepper in the Mekong Delta, Vietnam. *In vitro* screening revealed that both BP5 and BP103 possess a broad spectrum of biological activities, including the production of hydrolytic enzymes (protease, lipase, amylase, and cellulase) and iron mobilization via siderophores. These traits are known to support metabolic functions and iron competition (Maheshwari, 2013); however, their specific quantitative contribution to the *Xanthomonas* inhibition observed for BP5 and BP103 still requires further validation. Previous studies have consistently highlighted the efficacy of *Bacillus* strains in suppressing plant-associated bacterial pathogens (Marin et al., 2019; Zhou et al., 2023; Chandwani et al., 2024). Moreover, accumulating evidence further underscores *B. velezensis* as a species with substantial potential in integrated pest management (Rabbee et al., 2023). In future studies, *in vitro* and in planta assays could be conducted using cell-free extracts of hydrolytic enzymes and siderophores derived from BP5 and BP103 to more directly assess their potential role in antagonism against *Xanthomonas* pathogens. In addition, mutant strains lacking relevant genes could be generated to investigate the antagonistic role of these compounds.

Whole-genome analysis of BP5 and BP103 confirmed their taxonomic identity. Compared to the reference strains *B. velezensis* FZB42 and *B. subtilis* DSM10, the genomes of BP5 and BP103, in terms of length, G+C content, number of protein-coding genes, and prophage regions, appear more compact, with fewer predicted prophages and pseudogenes. Although this genomic pattern is consistent with a tendency toward increased genetic stability and a potential reduction in horizontal gene transfer events, additional experimental validation is still required. Moreover, whole-genome-based phylogenetic analysis placed BP5, BP103, and strain 160 (González-Léon et al., 2024) within the same clade, confirming their close genetic relatedness within *B. velezensis*.

Comparative genome analysis indicated that BP5 and BP103 share typical *B. velezensis* features, with genome sizes ranging from 3.9 to 4.2 Mb, G+C content of 46–47%, 3,900–4,200 protein-coding genes, and 2–19 prophage regions (Tang et al., 2025). These findings are consistent with reports for other strains such as K12 (Tang et al., 2025), S141 (Sibponkrung et al., 2017), EB14 (Sachin et al., 2021), and HNA3 (Zaid et al., 2022). In contrast, BP5 and BP103 possess fewer genes than FZB42 (4,231–4,337 genes) (Fan et al., 2018). The low numbers of predicted prophages (5 in BP5; 2 in BP103) indicate relatively compact genomes. These observations support the view that *B. velezensis* forms a relatively cohesive genetic group (Dunlap et al., 2016; Fan et al., 2017).

Moreover, Average Nucleotide Identity (ANI) analysis and orthologous gene comparison confirmed that ANI values for BP5 and BP103 against 25 other *B. velezensis* strains are all above 97%, unequivocally supporting their assignment to this species. Pan-genome analysis of 28 genomes, including the two study strains, *B. subtilis* DSM10, and 25 *B. velezensis* representatives, indicated with core-gene numbers decreasing as new genomes were added (Kadiri et al., 2023).

Besides, genomic functional analyses (COG, GO, KEGG, Pfam) showed that BP5 and BP103 have highly similar functional profiles. The genomic features of BP5, as inferred from functional annotation, are consistent with a relatively greater potential for genetic stability, secondary metabolite synthesis, and cell wall biogenesis, whereas BP103 generally encodes longer proteins with greater domain diversity, suggesting an enhanced potential for complex enzymes. Compared with other *B. velezensis* strains, BP5 and BP103 displayed functional profiles similar to HNA3 in amino acid transport/metabolism, transcription, carbohydrate metabolism, and secondary metabolite synthesis, while harboring fewer annotated genes in these categories (Zaid et al., 2022), indicating a more compact genetic repertoire. Similar to *B. velezensis* YC89 (Xie et al., 2023), and D103 (Yang et al., 2023), both strains also harbor a substantial number of genes with unknown function.

BP5, BP103, and the other *B. velezensis* strains in the dataset share 3,593 core gene families across the 28 genomes analyzed. Of these, 2,593 are present as single-copy orthologous genes in each genome, representing a conservative core genome, while the remaining core families include genes with multiple copies per genome. BP5 contains more multi-copy orthologs and unique orthologs than BP103, indicating higher genetic diversity in BP5. Among these core gene families, two functional groups, sporulation and secondary metabolites (NRPS/PKS), support biocontrol in *B. velezensis* (Zeriouh et al., 2014; Kadiri et al., 2023). Specifically, the spo0A_1 gene, which encodes Spo0A (and its promoter site 0A1) at loci BP5_CDS_1743 (BVE_01762) and BP103_CDS_2422 (BVE_02442), regulates sporulation initiation and may directly or indirectly control over 500 genes during the early stages of sporulation (Jun et al., 2025), while also influencing antagonism against other *Bacillus* strains (Shao et al., 2022). Recent genome and pan-genome studies have clarified the distribution of core, accessory, and unique genes across *B. velezensis* strains, providing a foundation for exploiting antagonistic traits to improve plant disease management through biocontrol bacteria (Kadiri et al., 2023).

Based on the unique genes, both BP5 and BP103 exhibit strong potential for antimicrobial peptide biosynthesis but with different characteristic configurations. In BP5, genes annotated as tyrocidine synthase 3 and as a subunit of a linear gramicidin–like synthetase system were identified based on sequence homology. However, tyrocidine and gramicidin are typically produced by *Brevibacillus brevis* and have not been reported in *B. velezensis*. Therefore, these annotations likely reflect homologous NRPS-related genes, and their functional roles require further experimental validation. These two types of peptides are typically produced by *Brevibacillus brevis*, contributing to broad-spectrum antibacterial and antifungal antagonistic activity (Nakano and Zuber, 1990; Yang et al., 2025). In contrast, BP103 possesses a diverse “reservoir” of biosynthetic genes, including those associated with the lantibiotic mersacidin and enzymes involved in its post-translational modification and maturation. In addition, genes related to linear gramicidin–like peptide synthesis and an indolmycin-related methyltransferase homolog, require confirmation by future comparative genomics and functional/metabolomic analyses.

In addition, secondary metabolite biosynthetic gene cluster (BGC) analysis revealed that both BP5 and BP103 possess 13 complete clusters encoding potent antimicrobial compounds, including surfactin (Gao et al., 2017), fengycin (Pajčin et al., 2020; Medeot et al., 2023), bacillaene (Patel et al., 1995; Butcher et al., 2007), macrolactin (Rabbee et al., 2019), difficidin (Chen et al., 2006, 2007; Wu et al., 2015), bacilysin (Wu et al., 2015; Özcengiz and Öğülür, 2015), bacillibactin (Miethke et al., 2006; Wen et al., 2011), mersacidin, and locillomycin. The repertoire of secondary metabolite biosynthetic gene clusters (BGCs) varies among *B. velezensis* strains and contributes to their antimicrobial activity (Palazzini et al., 2016). Although many BGCs are shared across the *Bacillus* genus, certain clusters are strain-specific within *B. velezensis* (Cai et al., 2017). The presence of clusters encoding difficidin, macrolactin, and bacillaen, macrolactin, and bacillaene gene clusters reaffirms the genetic signatures of *B. velezensis* (Fan et al., 2017; Sachin et al., 2021; González-Léon et al., 2024). Four unidentified terpene and PKS clusters could potentially contribute to antibacterial effects or other bioactive roles (Spantidos et al., 2025). They require isolation, extraction, and further inhibition assays in future studies.

Notably, both strains harbor distinct clusters for mersacidin and locillomycin (B/C), which are absent in strain FZB42 [lacking locillomycin (B/C)] (Herzner et al., 2011; Fan et al., 2018), and 160 (lacking mersacidin) (González-Léon et al., 2024). Mersacidin, a class II lanthipeptide, inhibits Gram-positive bacterial cell wall synthesis by binding lipid II and blocking glycosyl transfer during peptidoglycan formation (Brötz et al., 1995; Viel et al., 2021). Locillomycins are cyclic lipopeptides with potent antibacterial activity (Luo et al., 2015, 2019). Lipopeptides produced by related Bacillus and Virgibacillus strains that harbor locillomycin gene clusters show significant biosurfactant properties, strong reduction of oil–water interfacial tension and 100% emulsification index (Pereira et al., 2019; Essghaier et al., 2024). As no canonical, complete bacillomycin biosynthetic gene cluster was identified, BP5 and BP103 are considered to lack gene clusters for iturin family members (Cao et al., 2018; Choi et al., 2021; Zaid et al., 2022), as well as amylocyclicin, plantazolicin, kijanimicin, butirosin, and certain other bacteriocins reported in different *B. velezensis* strains (Chen L. et al., 2018; Wang K. X. et al., 2022; Zhou et al., 2023; Zaid et al., 2022), highlighting distinct secondary metabolite profiles among strains.

Regarding polymer degradation capacity, BP5 encodes 133 carbohydrate-active enzymes (CAZymes), only slightly more than the commercial strain FZB42 (129 genes), while BP103 has 129 genes, equivalent to FZB42. These enzymes cleave polysaccharide bonds in plant- and soil-derived polymers and enable utilization of diverse carbon sources, thereby contributing to nutrient acquisition in the rhizosphere environment, rather than directly lysing the peptidoglycan-based cell walls of bacterial pathogens (Hibbing et al., 2010). Biochemical assays independently confirmed the production of extracellular protease, lipase, amylase, cellulase, and siderophores, supporting the contribution of multiple enzymatic activities and nutritional competition mechanisms to antagonism, in addition to the CAZyme repertoire. Notably, BP5 exhibits a somewhat higher abundance of GH, CE, and CBM family genes than BP103, which may provide additional capacity for surface adhesion and carbohydrate turnover; however, the functional impact of this difference remains uncertain. Specifically, BP5 shows a net increase of four CAZyme modules relative to BP103 (GH +2, CE +1, CBM +1; Figure 8), mainly reflecting additional assignments to GH23, CE12, and CBM50. These families are consistent with potential enhancement of carbohydrate binding/adhesion (CBM50) and hydrolytic activity toward cell wall–associated or extracellular carbohydrate substrates (GH23/CE12), but such links are currently hypothetical and require experimental validation, and no direct competition assays with Xanthomonas have yet been performed.

Overall, the two *B. velezensis* strains BP5 and BP103 possess 13 biosynthetic gene clusters (BGCs) for secondary metabolites, which encode characteristic antimicrobial compounds capable of inhibiting plant pathogens. Moreover, BP5 is distinguished by 133 CAZymes (four more modules than BP103), which are consistent with potential hydrolytic activity toward carbohydrate substrates, as well as with enhanced rhizosphere adhesion and nutrient acquisition, although these ecological roles currently remain hypothetical and require experimental verification.

In future studies, the functions inferred from the CAZyme repertoire, secondary metabolite biosynthetic gene clusters (BGCs), and hydrolytic enzymes and siderophores of BP5 and BP103 should be experimentally validated. Specifically, they should be evaluated through *in vitro* and in planta assays using cell-free extracts, combined with the construction of mutant strains lacking the corresponding genes to clarify their roles in suppressing *Xanthomonas*. In parallel, metabolites encoded by the predicted BGCs, particularly tyrocidine/gramicidin-like clusters, should be identified by LC–MS analysis and bioassays to confirm their biosynthetic capacity and associated biological functions. Comprehensive genomic characterization of promising strains such as BP5 and BP103 will support the design of more precise disease management strategies and the development of safe, effective microbial-based agents; overall, these two strains represent promising candidates for managing bacterial leaf spot on pepper, with potential applications as bacterial biomass or as sources of bioactive metabolites.

## 5 Conclusion

This study comprehensively characterized the genomes of two *B. velezensis* strains, BP5 and BP103, isolated from the rhizosphere soil of peppers in the Mekong Delta, Vietnam. The results strongly confirm their promising biocontrol potential against *X. euvesicatoria*, the causal agent of BSP in peppers. Dual-culture antagonism assays revealed the largest inhibition zones for BP5, surpassing both BP103 and oxolinic acid. Both strains produce abundant extracellular enzymes (protease, lipase, amylase, cellulase) and siderophores, facilitating nutrient competition and nutrient competition.

Whole-genome sequencing demonstrated compact, genetically stable genomes with high nucleotide similarity to *B. velezensis*, most closely related to reference strain 160. Ortholog analysis showed BP5 enriched in multi-copy orthologs and unique paralogs; the pangenome further highlighted pronounced genetic divergence in BP5, suggesting potential for environmental adaptability. Notably, both strains encode numerous secondary metabolite biosynthetic gene clusters (BGCs), including BGCs encoding mersacidin and locillomycin (B/C) clusters, along with four previously undescribed terpene/PKS clusters. BP5 possesses a more abundant CAZyme repertoire than both BP103 and the commercial reference strain FZB42.

These findings establish BP5 and BP103 as highly promising, genetically stable biocontrol agents with diverse secondary metabolite profiles. Future studies should prioritize field efficacy trials and functional characterization of the novel bioactive compounds to fully realize their agricultural potential.

## Data Availability

The datasets presented in this study can be found in online repositories. The names of the repository/repositories and accession number(s) can be found at: https://www.ebi.ac.uk/ena/browser/view/GCA_977019115; https://www.ebi.ac.uk/ena/browser/view/GCA_977019105.

## Footnotes

1
https://proksee.ca/

2
http://www.ncbi.nlm.nih.gov/
