# Supplementary material for: Genomic characterization of Bacillus velezensis BP5 and BP103: insights into biocontrol potential against bacterial leaf spot on pepper
Source: Front Microbiol. 2026 Jul 21;17:1757903. doi: 10.3389/fmicb.2026.1757903 (PMC13433461; doi:10.3389/fmicb.2026.1757903)
Supplement: Supplementary file 1 [file Table_1.docx]

**Supplementary Materials**

**Supplementary Figure legends**

**Fig. S1.** Supplementary Figure S1. Whole‑genome phylogenetic tree of *Bacillus velezensis* strains. This tree shows the close evolutionary relationship of BP5 and BP103 with *B. velezensis* 160, with *B. subtilis* DSM10 included as an outgroup.

**Fig. S2.** Comparative COG, GO, and KEGG functional classifications of strains BP5 and BP103. A, C, E lần lượt là phân tích chức năng COG, GO, and KEGG từ bộ gen của dòng BP5; B, D, F lần lượt là phân tích chức năng COG, GO, and KEGG từ bộ gen của dòng BP103.

**Fig. S3.** Heatmap showing gene family multicopy–presence–absence (red/white/blue) and pangenome partitions (cloud, shell, and persistent) across the genomes of *B. velezensis* BP5, BP103, and related *B. velezensis* strains. The analysis highlights the dominance of persistent gene families and the high degree of genomic conservation between strains BP5 and 160.

**Supplementary Figure S1**

**Supplementary Figure S2**

**Supplementary Figure S3**
