# Supplementary material for: Genomic characterization of Bacillus velezensis BP5 and BP103: insights into biocontrol potential against bacterial leaf spot on pepper
Source: Front Microbiol. 2026 Jul 21;17:1757903. doi: 10.3389/fmicb.2026.1757903 (PMC13433461; doi:10.3389/fmicb.2026.1757903)
Supplement: Supplementary file 2 [file Table_2.docx]

**Supplementary Materials**

**Table S1.** Some functional genes differentially abundant between BP5 and BP103 identified according to COG

| **Replication/recombination/ repair+B4:M6** | | | | | **Secondary metabolites biosynthesis/transport/catabolism** | | | | | **Cell wall/membrane/envelope biogenesis** | | | | |
| --- | --- | --- | --- | --- | --- | --- | --- | --- | --- | --- | --- | --- | --- | --- |
| Families | more in BP5 | Name protein | Characteristics | Families | | more in BP5 | Name protein | Characteristics | Families | | more in BP5 | Name protein | Characteristics |  |
| 1042163.BRLA_c031450 | 1 | pi112 | PDDEXK-like domain of unknown function (DUF3799) | 326423.RBAM_003650; _003660 | | 2 | srfAA, srfAB | Catalyzes D‑alanylation of lipoteichoic acid, modulating Gram‑positive cell wall. | 1051501.AYTL01000028_gene2145 | | 1 | srtA | cell wall anchor domain |  |
| 1117379.BABA_17417 | 1 | - | Phage integrase family | 326423.RBAM_022030, _022000 | | 1 | - | Polyketide synthase of type I | 1178537.BA1_11289 | | 2 | xlyA | N-acetylmuramoyl-L-alanine amidase |  |
| 1121090.KB894727_gene1229 | 1 | - | Bacterial transcription activator, effector binding domain | 326423.RBAM_002630 | | 1 | yoaI | COG2368 Aromatic ring hydroxylase | 326423.RBAM_012470; 326423.RBAM_032770 | | 2 | xlyB; lytC | N-acetylmuramoyl-L-alanine amidase |  |
| 1122915.AUGY01000001_gene7239 | 1 | - | Recombinase | 326423.RBAM_018430, _018460 | | 2 | - | Catalyzes the first step in the D-alanylation of lipoteichoic acid (LTA) | 224308.BSU39230 | | 1 | wapA | COG3209 Rhs family protein |  |
| 1123405.AUMM01000039_gene156 | 1 | - | Phage integrase, N-terminal SAM-like domain | 720555.BATR1942_07350 | | 1 | - | Catalyzes the first step in the D-alanylation of lipoteichoic acid (LTA) | 326423.RBAM_032810 | | 1 | mnaA | Belongs to the UDP-N-acetylglucosamine 2-epimerase family |  |
| 1178537.BA1_15559 | 1 | int7 | Belongs to the 'phage' integrase family | 1051501.AYTL01000030_gene2250 | | 1 | ykvO | Enoyl-(Acyl carrier protein) reductase | 326423.RBAM_032820 | | 1 | gtaB | UTP-glucose-1-phosphate uridylyltransferase |  |
| 1236976.JCM16418_4600 | 1 | - | Recombinase zinc beta ribbon domain | 326423.RBAM_037840 | | 1 | bdh | Catalyzes the formation of acetoacetate from 3-hydroxybutyrate | 326423.RBAM_032830 | | 1 | tagH | Part of the ABC transporter complex TagGH involved in teichoic acids export. Responsible for energy coupling to the transport system |  |
| 1280676.AUJO01000007_gene2148 | 1 | - | SNF2 family N-terminal domain | 326423.RBAM_006080 | | 1 | - | ubiE/COQ5 methyltransferase family | 326423.RBAM_032840 | | 1 | tagG | Transport permease protein |  |
| 1280692.AUJL01000004_gene859 | 1 | - | GIY-YIG type nucleases (URI domain) | 326423.RBAM_007760 | | 1 | - | calcium- and calmodulin-responsive adenylate cyclase activity | 326423.RBAM_032850 | | 1 | tagF | glycosyl glycerophosphate transferases involved in teichoic acid biosynthesis TagF TagB EpsJ RodC |  |
| 1347369.CCAD010000078_gene3010 | 1 | - | Excalibur calcium-binding domain | 1051501.AYTL01000011_gene162 | | 1 | - | Collagen triple helix repeat (20 copies) |  | |  |  |  |  |
| 1166018.FAES_4010, 224308.BSU20360 | 2 | - | Participates in initiation and elongation during chromosome replication, DNA primase activity |  | |  |  |  |  | |  |  |  |  |
| 1450694.BTS2_3308, 649639.Bcell_1133 | 2 | - | Domain of unknown function (DUF4942), Uncharacterized conserved protein (DUF2075) |  | |  |  |  |  | |  |  |  |  |
| 224308.BSU20500, 649747.HMPREF0083_05930 | 2 | ligB | ATP-dependent DNA ligase, DNA polymerase |  | |  |  |  |  | |  |  |  |  |
| 224308.BSU26260, BSU26270, 326423.RBAM_018830, 720555.BATR1942_07860 | 4 | yqaM, yqaL | IstB-like ATP binding protein, DnaD domain protein, HNH/ENDO VII nuclease, Histone-like DNA-binding protein |  | |  |  |  |  | |  |  |  |  |
| 224308.BSU21500, 224308.BSU26250, 279010.BL00985 | 3 | dinB2, rusA, yqaS | DNA polymerase type‑Y, Pol IV; Endodeoxyribonuclease RusA; DNA packaging |  | |  |  |  |  | |  |  |  |  |
| 1384057.CD33_12320, 224308.BSU20810 | 2 | - | Belongs to the 'phage' integrase family |  | |  |  |  |  | |  |  |  |  |
| 1444310.JANV01000205_gene606 | 1 | - | GIY-YIG catalytic domain |  | |  |  |  |  | |  |  |  |  |
|  |  |  |  |  | |  |  |  |  | |  |  |  |  |
| 224308.BSU19830 | 1 | - | Endonuclease resolving Holliday junctions for DNA repair and segregation. |  | |  |  |  |  | |  |  |  |  |
| 224308.BSU20350 | 1 | - | Single-stranded-DNA-specific exonuclease RecJ |  | |  |  |  |  | |  |  |  |  |
| 224308.BSU20390 | 1 | - | AAA domain |  | |  |  |  |  | |  |  |  |  |
| 224308.BSU20390 | 1 | - | AAA domain |  | |  |  |  |  | |  |  |  |  |

**Table S2.** Candidate Large Protein Differences (>2000 aa) Between BP5 and BP103

| **Protein group** | **BP5** | **BP103** | **Simple interpretation** |
| --- | --- | --- | --- |
| Bacillomycin BamB-like | NRPS/PKS; 5362 aa; 120 signatures | bamB; 5363 aa; 121 signatures | Almost the same size; mainly annotation-name difference. |
| Bacillomycin BamC | Not clearly annotated | bamC; 2622 aa; 64 signatures | Clearly annotated in BP103 only. |
| Additional large NRPS | Not clearly detected | NRPS; 3591 aa; 87 signatures | Candidate BP103-specific large NRPS. |
| DNRLRE domain protein | DNRLRE domain protein; 2343 aa; 21 signatures | Not clearly detected | Candidate BP5-specific large protein. |
| NTTRR-F1 domain protein | Not clearly detected | NTTRR-F1 domain; 2294 aa; 16 signatures | Candidate BP103-specific large protein. |

**Table S3.** The number genes of orthogroup from twenty seven *Bacillus velezensis* and *Bacillus subtilis* DSM10

| **Species** | **Single_copy** | **Multi_copy** | **Other** | **Unique_paralogs** |
| --- | --- | --- | --- | --- |
| *Bacillus subtilis DSM10* | *2593* | *411* | *631* | *30* |
| *Bacillus velezensis* 10075 | 2593 | 441 | 1002 | 2 |
| *Bacillus velezensis* 11640 | 2593 | 431 | 1076 | 1 |
| *Bacillus velezensis* 12Y | 2593 | 461 | 1072 | 0 |
| *Bacillus velezensis* 160 | 2593 | 413 | 968 | 0 |
| *Bacillus velezensis* 77-E7-A5 | 2593 | 375 | 875 | 0 |
| *Bacillus velezensis* 77-E7-BHI | 2593 | 376 | 874 | 0 |
| *Bacillus velezensis* 77-E7 | 2593 | 376 | 874 | 0 |
| *Bacillus velezensis* AP46 | 2593 | 397 | 994 | 1 |
| *Bacillus velezensis* BP1 | 2593 | 381 | 790 | 1 |
| *Bacillus velezensis* BP103 | 2593 | 336 | 763 | 1 |
| *Bacillus velezensis* BP5 | 2593 | 388 | 910 | 5 |
| *Bacillus velezensis* CBMB205 | 2593 | 303 | 692 | 1 |
| *Bacillus velezensis* CIMT3 | 2593 | 416 | 999 | 0 |
| *Bacillus velezensis* DSYZ | 2593 | 416 | 1058 | 0 |
| *Bacillus velezensis* FS3 | 2593 | 328 | 741 | 0 |
| *Bacillus velezensis* FZB42 | 2593 | 290 | 782 | 0 |
| *Bacillus velezensis* GJJK74 | 2593 | 417 | 1003 | 0 |
| *Bacillus velezensis* ID-A03 | 2593 | 416 | 1015 | 0 |
| *Bacillus velezensis* ID-A04 | 2593 | 417 | 1018 | 0 |
| *Bacillus velezensis* LOH112 | 2593 | 462 | 1038 | 3 |
| *Bacillus velezensis* Lzh-a42 | 2593 | 437 | 1000 | 0 |
| *Bacillus velezensis* PD9 | 2593 | 410 | 1038 | 0 |
| *Bacillus velezensis* PEBA20 | 2593 | 443 | 956 | 0 |
| *Bacillus velezensis* SRCM123422 | 2593 | 453 | 1011 | 0 |
| *Bacillus velezensis* TZS01 | 2593 | 410 | 958 | 0 |
| *Bacillus velezensis* XRD006 | 2593 | 457 | 1069 | 0 |
| *Bacillus velezensis* YJ11-1-4 | 2593 | 315 | 619 | 0 |
| TOTAL orthogroups | 2593 | 668 | 2093 | 45 |

**Table S4.** Functional differences in unique genes between BP5 and BP103

| **Functional group** | **BP5-only (456 families)** | **Percentage in unique genes (%)** | **BP103-only (203 families)** | **Percentage in unique genes (%)** |
| --- | --- | --- | --- | --- |
| Hypothetical/Uncharacterized Proteins | 365 | 80.04 | 132 | 65.02 |
| Genetic Information Processing | 28 | 6.14 | 13 | 6.40 |
| Metabolism/ Secondary Metabolites | 23 | 5.04 | 15 | 7.39 |
| Signal Transduction/ Defense Mechanisms | 18 | 3.95 | 22 | 10.84 |
| Cellular Processes/ Transport | 17 | 3.73 | 20 | 9.85 |
| Mobile Genetic Elements/ Phages | 5 | 1.10 | 1 | 0.49 |
| **Total** | **456** | **100** | **203** | **100** |

**Table S5.** Details of unique genes present in *B. velezensis* strain BP5 and BP103

| **BP5** | | | | | | | **BP103** | | | | | | |
| --- | --- | --- | --- | --- | --- | --- | --- | --- | --- | --- | --- | --- | --- |
| No | Gene family (rep) | Partition | Annotation | Gene IDs (BP5) | Locus tags | n gene | No | Gene family (rep) | Partition | Annotation | Gene IDs (BP5) | Locus tags | n gene |
| 1 | BP5_CDS_3182 | shell | 4-hydroxy-tetrahydrodipicolinate synthase | BP5_CDS_3182 | BVE_03218 | 1 | 1 | BP103_CDS_3005 | persistent | 4-hydroxy-tetrahydrodipicolinate synthase | BP103_CDS_3005 | BVE_03036 | 1 |
| 2 | BP5_CDS_3728 | shell | Aerobic respiration control sensor protein ArcB | BP5_CDS_3728 | BVE_03773 | 1 | 2 | BP103_CDS_1293 | persistent | 5-amino-6-(5-phospho-D-ribitylamino)uracil phosphatase YwtE | BP103_CDS_1293 | BVE_01307 | 1 |
| 3 | BP5_CDS_1345 | shell | Alkaline phosphatase synthesis transcriptional regulatory protein SphR | BP5_CDS_1345 | BVE_01353 | 1 | 3 | BP103_CDS_1839 | persistent | ATP-dependent DNA helicase Rep | BP103_CDS_1839 | BVE_01854 | 1 |
| 4 | BP5_CDS_0524 | shell | Amidase enhancer | BP5_CDS_0524 | BVE_00529 | 1 | 4 | BP103_CDS_1910 | persistent | Acetyltransferase | BP103_CDS_1910 | BVE_01929 | 1 |
| 5 | BP5_CDS_2882 | shell | Antitoxin EndoAI | BP5_CDS_2882 | BVE_02911 | 1 | 5 | BP103_CDS_1433 | persistent | Amidase enhancer | BP103_CDS_1433 | BVE_01448 | 1 |
| 6 | BP5_CDS_3448 | persistent | Aryl-phospho-beta-D-glucosidase BglC | BP5_CDS_3448 | BVE_03486 | 1 | 6 | BP103_CDS_0749 | persistent | Arginine exporter protein ArgO | BP103_CDS_0749 | BVE_00751 | 1 |
| 7 | BP5_CDS_2861 | shell | Bis(5'-nucleosyl)-tetraphosphatase, symmetrical | BP5_CDS_2861 | BVE_02890 | 1 | 7 | BP103_CDS_1125 | persistent | Arsenite methyltransferase | BP103_CDS_1125 | BVE_01138 | 1 |
| 8 | BP5_CDS_1382 | persistent | Bleomycin resistance protein | BP5_CDS_1382 | BVE_01390 | 1 | 8 | BP103_CDS_1291 | persistent | Catabolite control protein A | BP103_CDS_1291 | BVE_01305 | 1 |
| 9 | BP5_CDS_2898 | shell | Cell wall-binding protein YocH | BP5_CDS_2898 | BVE_02927 | 1 | 9 | BP103_CDS_2999 | persistent | D,L-glycerol 3-phosphate phosphatase | BP103_CDS_2999 | BVE_03030 | 1 |
| 10 | BP5_CDS_1011 | shell | Chromosomal replication initiator protein DnaA | BP5_CDS_1011 | BVE_01019 | 1 | 10 | BP103_CDS_3446 | persistent | Daunorubicin/doxorubicin resistance ATP-binding protein DrrA | BP103_CDS_3446 | BVE_03491 | 1 |
| 11 | BP5_CDS_3161 | shell | Copper-sensing transcriptional repressor CsoR | BP5_CDS_3161 | BVE_03197 | 1 | 11 | BP103_CDS_1968 | persistent | Exo-glucosaminidase LytG | BP103_CDS_1968 | BVE_01987 | 1 |
| 12 | BP5_CDS_0046 | shell | D-beta-hydroxybutyrate dehydrogenase | BP5_CDS_0046 | BVE_00047 | 1 | 12 | BP103_CDS_2575 | persistent | Glyoxal reductase | BP103_CDS_2575 | BVE_02596 | 1 |
| 13 | BP5_CDS_2899 | shell | DNA polymerase III subunit alpha | BP5_CDS_2899 | BVE_02928 | 1 | 13 | BP103_CDS_0748 | persistent | HTH-type transcriptional regulator NorG | BP103_CDS_0748 | BVE_00750 | 1 |
| 14 | BP5_CDS_3428 | shell | DNA polymerase IV | BP5_CDS_3428 | BVE_03466 | 1 | 14 | BP103_CDS_2009 | persistent | HTH-type transcriptional regulator SinR | BP103_CDS_2009 | BVE_02028 | 1 |
| 15 | BP5_CDS_2985 | shell | DNA-binding protein HU | BP5_CDS_2985 | BVE_03014 | 1 | 15 | BP103_CDS_2968 | persistent | Haloacetate dehalogenase H-1 | BP103_CDS_2968 | BVE_02999 | 1 |
| 16 | BP5_CDS_3647 | shell | Daunorubicin/doxorubicin resistance ATP-binding protein DrrA | BP5_CDS_3647 | BVE_03690 | 1 | 16 | BP103_CDS_3000 | persistent | Hydroxymethylpyrimidine/phosphomethylpyrimidine kinase | BP103_CDS_3000 | BVE_03031 | 1 |
| 17 | BP5_CDS_2917 | shell | Deoxyuridine 5'-triphosphate nucleotidohydrolase | BP5_CDS_2917 | BVE_02946 | 1 | 17 | BP103_CDS_1811 | persistent | Lactococcin-G-processing and transport ATP-binding protein LagD | BP103_CDS_1811 | BVE_01826 | 1 |
| 18 | BP5_CDS_2869 | shell | Dihydrofolate reductase type 3 | BP5_CDS_2869 | BVE_02898 | 1 | 18 | BP103_CDS_1807 | persistent | Lantibiotic mersacidin | BP103_CDS_1807 | BVE_01822 | 1 |
| 19 | BP5_CDS_1038 | shell | Exochitinase 1 | BP5_CDS_1038 | BVE_01046 | 1 | 19 | BP103_CDS_1389 | persistent | Linear gramicidin dehydrogenase LgrE | BP103_CDS_1389 | BVE_01404 | 1 |
| 20 | BP5_CDS_0041 | shell | FMN-dependent NADH-azoreductase 1 | BP5_CDS_0041 | BVE_00042 | 1 | 20 | BP103_CDS_0028 | persistent | Linear gramicidin synthase subunit D | BP103_CDS_0028 | BVE_00029 | 1 |
| 21 | BP5_CDS_3152 | persistent | General stress protein 14 | BP5_CDS_3152 | BVE_03188 | 1 | 21 | BP103_CDS_3535 | persistent | Mannuronan C5-epimerase AlgE5 | BP103_CDS_3535 | BVE_03580 | 1 |
| 22 | BP5_CDS_2907 | shell | Glutamyl endopeptidase | BP5_CDS_2907 | BVE_02936 | 1 | 22 | BP103_CDS_1434 | persistent | Membrane-bound protein LytA | BP103_CDS_1434 | BVE_01449 | 1 |
| 23 | BP5_CDS_2226 | persistent | Glycerophosphodiester phosphodiesterase | BP5_CDS_2226 | BVE_02248 | 1 | 23 | BP103_CDS_1809 | persistent | Mersacidin decarboxylase | BP103_CDS_1809 | BVE_01824 | 1 |
| 24 | BP5_CDS_3996 | shell | Group II intron-encoded protein LtrA | BP5_CDS_3996 | BVE_04044 | 1 | 24 | BP103_CDS_1841 | persistent | Mrr restriction system protein | BP103_CDS_1841 | BVE_01856 | 1 |
| 25 | BP5_CDS_0047 | shell | HTH-type transcriptional regulator CynR | BP5_CDS_0047 | BVE_00048 | 1 | 25 | BP103_CDS_1919 | persistent | Multidrug-efflux transporter 1 regulator | BP103_CDS_1919 | BVE_01938 | 1 |
| 26 | BP5_CDS_0039 | shell | HTH-type transcriptional regulator ImmR | BP5_CDS_0039 | BVE_00040 | 1 | 26 | BP103_CDS_1914 | persistent | Multiple antibiotic resistance protein MarR | BP103_CDS_1914 | BVE_01933 | 1 |
| 27 | BP5_CDS_0245 | persistent | HTH-type transcriptional regulator MhqR | BP5_CDS_0245 | BVE_00250 | 1 | 27 | BP103_CDS_1432 | persistent | N-acetylmuramoyl-L-alanine amidase LytC | BP103_CDS_1432 | BVE_01447 | 1 |
| 28 | BP5_CDS_1000 | shell | HTH-type transcriptional regulator Xre | BP5_CDS_1000 | BVE_01008 | 1 | 28 | BP103_CDS_1962 | persistent | N-acetylmuramoyl-L-alanine amidase XlyA | BP103_CDS_1962 | BVE_01981 | 1 |
| 29 | BP5_CDS_3164 | shell | Hydroxyacylglutathione hydrolase GloC | BP5_CDS_3164 | BVE_03200 | 1 | 29 | BP103_CDS_3001 | persistent | N-demethylindolmycin N-methyltransferase | BP103_CDS_3001 | BVE_03032 | 1 |
| 30 | BP5_CDS_1336 | shell | Lactococcin-G-processing and transport ATP-binding protein LagD | BP5_CDS_1336 | BVE_01344 | 1 | 30 | BP103_CDS_0709 | persistent | Oligopeptide-binding protein AppA | BP103_CDS_0709 | BVE_00711 | 1 |
| 31 | BP5_CDS_2287 | persistent | Linear gramicidin synthase subunit D | BP5_CDS_2287 | BVE_02315 | 1 | 31 | BP103_CDS_3016 | persistent | Oxalate:formate antiporter | BP103_CDS_3016 | BVE_03047 | 1 |
| 32 | BP5_CDS_3995 | shell | Membrane-bound lytic murein transglycosylase C | BP5_CDS_3995 | BVE_04043 | 1 | 32 | BP103_CDS_1607 | persistent | Phosphatase RapF inhibitor | BP103_CDS_1607 | BVE_01622 | 1 |
| 33 | BP5_CDS_0523 | shell | Membrane-bound protein LytA | BP5_CDS_0523 | BVE_00528 | 1 | 33 | BP103_CDS_1802 | persistent | Phosphate regulon sensor protein PhoR | BP103_CDS_1802 | BVE_01817 | 1 |
| 34 | BP5_CDS_2222 | persistent | Multidrug resistance protein Stp | BP5_CDS_2222 | BVE_02244 | 1 | 34 | BP103_CDS_1441 | persistent | Poly(glycerol-phosphate) alpha-glucosyltransferase | BP103_CDS_1441 | BVE_01456 | 1 |
| 35 | BP5_CDS_2915 | shell | Multifunctional non-homologous end joining protein LigD | BP5_CDS_2915 | BVE_02944 | 1 | 35 | BP103_CDS_1435 | persistent | Polyisoprenyl-teichoic acid--peptidoglycan teichoic acid transferase TagU | BP103_CDS_1435 | BVE_01450 | 1 |
| 36 | BP5_CDS_1049 | shell | N-acetylmuramoyl-L-alanine amidase CwlA | BP5_CDS_1049 | BVE_01057 | 1 | 36 | BP103_CDS_1840 | persistent | Protease HtpX | BP103_CDS_1840 | BVE_01855 | 1 |
| 37 | BP5_CDS_3435 | shell | N-acetylmuramoyl-L-alanine amidase CwlA | BP5_CDS_3435 | BVE_03473 | 1 | 37 | BP103_CDS_2573 | persistent | Putative racemase YgeA | BP103_CDS_2573 | BVE_02594 | 1 |
| 38 | BP5_CDS_0525 | shell | N-acetylmuramoyl-L-alanine amidase LytC | BP5_CDS_0525 | BVE_00530 | 1 | 38 | BP103_CDS_1959 | persistent | Putative ribonuclease YeeF | BP103_CDS_1959 | BVE_01978 | 1 |
| 39 | BP5_CDS_1028 | shell | NAD(+)--arginine ADP-ribosyltransferase EFV | BP5_CDS_1028 | BVE_01036 | 1 | 39 | BP103_CDS_3021 | persistent | RNA polymerase-binding transcription factor CarD | BP103_CDS_3021 | BVE_03052 | 1 |
| 40 | BP5_CDS_1446 | persistent | Oligopeptide-binding protein AppA | BP5_CDS_1446 | BVE_01455 | 1 | 40 | BP103_CDS_1606 | persistent | Response regulator aspartate phosphatase F | BP103_CDS_1606 | BVE_01621 | 1 |
| 41 | BP5_CDS_0522 | shell | Polyisoprenyl-teichoic acid--peptidoglycan teichoic acid transferase TagU | BP5_CDS_0522 | BVE_00527 | 1 | 41 | BP103_CDS_0972 | persistent | Response regulator aspartate phosphatase H | BP103_CDS_0972 | BVE_00984 | 1 |
| 42 | BP5_CDS_2881 | shell | Protein NrdI | BP5_CDS_2881 | BVE_02910 | 1 | 42 | BP103_CDS_3033 | persistent | Response regulator aspartate phosphatase I | BP103_CDS_3033 | BVE_03064 | 1 |
| 43 | BP5_CDS_1054 | shell | Protein-export protein SecB | BP5_CDS_1054 | BVE_01062 | 1 | 43 | BP103_CDS_3034 | persistent | Response regulator aspartate phosphatase I | BP103_CDS_3034 | BVE_03065 | 1 |
| 44 | BP5_CDS_0040 | shell | Putative HTH-type transcriptional regulator | BP5_CDS_0040 | BVE_00041 | 1 | 44 | BP103_CDS_1119 | persistent | Sporulation delaying protein C | BP103_CDS_1119 | BVE_01132 | 1 |
| 45 | BP5_CDS_2914 | shell | Putative SOS response-associated peptidase YedK | BP5_CDS_2914 | BVE_02943 | 1 | 45 | BP103_CDS_1121 | persistent | Sporulation-delaying protein SdpA | BP103_CDS_1121 | BVE_01134 | 1 |
| 46 | BP5_CDS_2900 | shell | Putative SPBc2 prophage-derived single-strand DNA-specific exonuclease YorK | BP5_CDS_2900 | BVE_02929 | 1 | 46 | BP103_CDS_1120 | persistent | Sporulation-delaying protein SdpB | BP103_CDS_1120 | BVE_01133 | 1 |
| 47 | BP5_CDS_1442 | persistent | Putative bacilysin exporter BacE | BP5_CDS_1442 | BVE_01451 | 1 | 47 | BP103_CDS_3434 | persistent | Sulfate transport system permease protein CysT | BP103_CDS_3434 | BVE_03479 | 1 |
| 48 | BP5_CDS_0516 | shell | Putative teichuronic acid biosynthesis glycosyltransferase TuaG | BP5_CDS_0516 | BVE_00521 | 1 | 48 | BP103_CDS_3435 | persistent | Sulfate transport system permease protein CysW | BP103_CDS_3435 | BVE_03480 | 1 |
| 49 | BP5_CDS_0038 | shell | Quaternary ammonium compound-resistance protein QacC | BP5_CDS_0038 | BVE_00039 | 1 | 49 | BP103_CDS_3433 | persistent | Sulfate-binding protein | BP103_CDS_3433 | BVE_03478 | 1 |
| 50 | BP5_CDS_1074 | shell | Response regulator aspartate phosphatase A | BP5_CDS_1074 | BVE_01082 | 1 | 50 | BP103_CDS_3436 | persistent | Sulfate/thiosulfate import ATP-binding protein CysA | BP103_CDS_3436 | BVE_03481 | 1 |
| 51 | BP5_CDS_0354 | shell | Response regulator aspartate phosphatase F | BP5_CDS_0354 | BVE_00359 | 1 | 51 | BP103_CDS_1915 | persistent | Sulfurtransferase | BP103_CDS_1915 | BVE_01934 | 1 |
| 52 | BP5_CDS_3430 | shell | Response regulator aspartate phosphatase I | BP5_CDS_3430 | BVE_03468 | 1 | 52 | BP103_CDS_1440 | persistent | Teichoic acid poly(glycerol phosphate) polymerase | BP103_CDS_1440 | BVE_01455 | 1 |
| 53 | BP5_CDS_2894 | shell | Ribose-phosphate pyrophosphokinase | BP5_CDS_2894 | BVE_02923 | 1 | 53 | BP103_CDS_1439 | persistent | Teichoic acid translocation permease protein TagG | BP103_CDS_1439 | BVE_01454 | 1 |
| 54 | BP5_CDS_3184 | shell | Ribosomal RNA large subunit methyltransferase Cfr | BP5_CDS_3184 | BVE_03220 | 1 | 54 | BP103_CDS_1438 | persistent | Teichoic acids export ATP-binding protein TagH | BP103_CDS_1438 | BVE_01453 | 1 |
| 55 | BP5_CDS_2793 | shell | SPBc2 prophage-derived uncharacterized protein YolA | BP5_CDS_2793 | BVE_02822 | 1 | 55 | BP103_CDS_2019 | persistent | Threonine--tRNA ligase 2 | BP103_CDS_2019 | BVE_02039 | 1 |
| 56 | BP5_CDS_2935 | shell | SPBc2 prophage-derived uncharacterized protein YopX | BP5_CDS_2935 | BVE_02964 | 1 | 56 | BP103_CDS_3002 | persistent | Thymidylate synthase | BP103_CDS_3002 | BVE_03033 | 1 |
| 57 | BP5_CDS_2897 | shell | SPBc2 prophage-derived uncharacterized protein YorP | BP5_CDS_2897 | BVE_02926 | 1 | 57 | BP103_CDS_1808 | persistent | Transcriptional regulatory protein OmpR | BP103_CDS_1808 | BVE_01823 | 1 |
| 58 | BP5_CDS_2895 | shell | SPBc2 prophage-derived uncharacterized protein YorR | BP5_CDS_2895 | BVE_02924 | 1 | 58 | BP103_CDS_1803 | persistent | Transcriptional regulatory protein SrrA | BP103_CDS_1803 | BVE_01818 | 1 |
| 59 | BP5_CDS_1340 | shell | Sensor histidine kinase ComP | BP5_CDS_1340 | BVE_01348 | 1 | 59 | BP103_CDS_2013 | persistent | Transposase from transposon Tn916 | BP103_CDS_2013 | BVE_02032 | 1 |
| 60 | BP5_CDS_1344 | shell | Sensor histidine kinase CssS | BP5_CDS_1344 | BVE_01352 | 1 | 60 | BP103_CDS_3352 | persistent | Type-1 restriction enzyme R protein | BP103_CDS_3352 | BVE_03397 | 1 |
| 61 | BP5_CDS_3729 | shell | Sensory transduction protein LytR | BP5_CDS_3729 | BVE_03774 | 1 | 61 | BP103_CDS_1991 | persistent | Tyrosine recombinase XerD | BP103_CDS_1991 | BVE_02010 | 1 |
| 62 | BP5_CDS_3162 | shell | Sulfur carrier protein DsrE2 | BP5_CDS_3162 | BVE_03198 | 1 | 62 | BP103_CDS_0144 | persistent | hypothetical protein | BP103_CDS_0144 | BVE_00145 | 1 |
| 63 | BP5_CDS_3163 | shell | Sulfur carrier protein TusA | BP5_CDS_3163 | BVE_03199 | 1 | 63 | BP103_CDS_0145 | persistent | hypothetical protein | BP103_CDS_0145 | BVE_00146 | 1 |
| 64 | BP5_CDS_3165 | shell | Sulfur carrier protein TusA | BP5_CDS_3165 | BVE_03201 | 1 | 64 | BP103_CDS_0146 | persistent | hypothetical protein | BP103_CDS_0146 | BVE_00147 | 1 |
| 65 | BP5_CDS_0517 | shell | Teichoic acid poly(glycerol phosphate) polymerase | BP5_CDS_0517 | BVE_00522 | 1 | 65 | BP103_CDS_0147 | persistent | hypothetical protein | BP103_CDS_0147 | BVE_00148 | 1 |
| 66 | BP5_CDS_0518 | shell | Teichoic acid translocation permease protein TagG | BP5_CDS_0518 | BVE_00523 | 1 | 66 | BP103_CDS_0148 | persistent | hypothetical protein | BP103_CDS_0148 | BVE_00149 | 1 |
| 67 | BP5_CDS_0519 | shell | Teichoic acids export ATP-binding protein TagH | BP5_CDS_0519 | BVE_00524 | 1 | 67 | BP103_CDS_0290 | persistent | hypothetical protein | BP103_CDS_0290 | BVE_00291 | 1 |
| 68 | BP5_CDS_2877 | shell | Thioredoxin | BP5_CDS_2877 | BVE_02906 | 1 | 68 | BP103_CDS_0479 | persistent | hypothetical protein | BP103_CDS_0479 | BVE_00480 | 1 |
| 69 | BP5_CDS_2667 | persistent | Threonine--tRNA ligase 1 | BP5_CDS_2667 | BVE_02696 | 1 | 69 | BP103_CDS_0573 | persistent | hypothetical protein | BP103_CDS_0573 | BVE_00574 | 1 |
| 70 | BP5_CDS_2870 | shell | Thymidylate synthase 1 | BP5_CDS_2870 | BVE_02899 | 1 | 70 | BP103_CDS_0574 | persistent | hypothetical protein | BP103_CDS_0574 | BVE_00575 | 1 |
| 71 | BP5_CDS_1339 | shell | Transcriptional regulatory protein ComA | BP5_CDS_1339 | BVE_01347 | 1 | 71 | BP103_CDS_0651 | persistent | hypothetical protein | BP103_CDS_0651 | BVE_00652 | 1 |
| 72 | BP5_CDS_2221 | persistent | Transcriptional repressor SdpR | BP5_CDS_2221 | BVE_02243 | 1 | 72 | BP103_CDS_0779 | persistent | hypothetical protein | BP103_CDS_0779 | BVE_00781 | 1 |
| 73 | BP5_CDS_2978 | shell | Tubulin-like protein CetZ | BP5_CDS_2978 | BVE_03007 | 1 | 73 | BP103_CDS_0788 | persistent | hypothetical protein | BP103_CDS_0788 | BVE_00790 | 1 |
| 74 | BP5_CDS_2286 | persistent | Tyrocidine synthase 3 | BP5_CDS_2286 | BVE_02314 | 1 | 74 | BP103_CDS_0954 | persistent | hypothetical protein | BP103_CDS_0954 | BVE_00957 | 1 |
| 75 | BP5_CDS_0996 | shell | Tyrosine recombinase XerC | BP5_CDS_0996 | BVE_01004 | 1 | 75 | BP103_CDS_0969 | persistent | hypothetical protein | BP103_CDS_0969 | BVE_00981 | 1 |
| 76 | BP5_CDS_2943 | shell | Tyrosine recombinase XerC | BP5_CDS_2943 | BVE_02972 | 1 | 76 | BP103_CDS_0970 | persistent | hypothetical protein | BP103_CDS_0970 | BVE_00982 | 1 |
| 77 | BP5_CDS_3009 | shell | Tyrosine recombinase XerC | BP5_CDS_3009 | BVE_03038 | 1 | 77 | BP103_CDS_0971 | persistent | hypothetical protein | BP103_CDS_0971 | BVE_00983 | 1 |
| 78 | BP5_CDS_0867 | shell | Tyrosine recombinase XerD | BP5_CDS_0867 | BVE_00874 | 1 | 78 | BP103_CDS_0973 | persistent | hypothetical protein | BP103_CDS_0973 | BVE_00985 | 1 |
| 79 | BP5_CDS_0869 | shell | Tyrosine recombinase XerD | BP5_CDS_0869 | BVE_00876 | 1 | 79 | BP103_CDS_0974 | persistent | hypothetical protein | BP103_CDS_0974 | BVE_00986 | 1 |
| 80 | BP5_CDS_2655 | shell | Tyrosine recombinase XerD | BP5_CDS_2655 | BVE_02683 | 1 | 80 | BP103_CDS_1106 | persistent | hypothetical protein | BP103_CDS_1106 | BVE_01119 | 1 |
| 81 | BP5_CDS_0244 | persistent | Tyrosine--tRNA ligase | BP5_CDS_0244 | BVE_00249 | 1 | 81 | BP103_CDS_1126 | persistent | hypothetical protein | BP103_CDS_1126 | BVE_01139 | 1 |
| 82 | BP5_CDS_0034 | shell | hypothetical protein | BP5_CDS_0034 | BVE_00035 | 1 | 82 | BP103_CDS_1236 | persistent | hypothetical protein | BP103_CDS_1236 | BVE_01249 | 1 |
| 83 | BP5_CDS_0035 | shell | hypothetical protein | BP5_CDS_0035 | BVE_00036 | 1 | 83 | BP103_CDS_1237 | persistent | hypothetical protein | BP103_CDS_1237 | BVE_01250 | 1 |
| 84 | BP5_CDS_0036 | shell | hypothetical protein | BP5_CDS_0036 | BVE_00037 | 1 | 84 | BP103_CDS_1284 | persistent | hypothetical protein | BP103_CDS_1284 | BVE_01298 | 1 |
| 85 | BP5_CDS_0048 | shell | hypothetical protein | BP5_CDS_0048 | BVE_00049 | 1 | 85 | BP103_CDS_1290 | persistent | hypothetical protein | BP103_CDS_1290 | BVE_01304 | 1 |
| 86 | BP5_CDS_0049 | shell | hypothetical protein | BP5_CDS_0049 | BVE_00050 | 1 | 86 | BP103_CDS_1292 | persistent | hypothetical protein | BP103_CDS_1292 | BVE_01306 | 1 |
| 87 | BP5_CDS_0050 | shell | hypothetical protein | BP5_CDS_0050 | BVE_00051 | 1 | 87 | BP103_CDS_1328 | persistent | hypothetical protein | BP103_CDS_1328 | BVE_01342 | 1 |
| 88 | BP5_CDS_0104 | persistent | hypothetical protein | BP5_CDS_0104 | BVE_00109 | 1 | 88 | BP103_CDS_1346 | persistent | hypothetical protein | BP103_CDS_1346 | BVE_01361 | 1 |
| 89 | BP5_CDS_0108 | shell | hypothetical protein | BP5_CDS_0108 | BVE_00113 | 1 | 89 | BP103_CDS_1491 | persistent | hypothetical protein | BP103_CDS_1491 | BVE_01506 | 1 |
| 90 | BP5_CDS_0109 | shell | hypothetical protein | BP5_CDS_0109 | BVE_00114 | 1 | 90 | BP103_CDS_1524 | persistent | hypothetical protein | BP103_CDS_1524 | BVE_01539 | 1 |
| 91 | BP5_CDS_0110 | shell | hypothetical protein | BP5_CDS_0110 | BVE_00115 | 1 | 91 | BP103_CDS_1706 | persistent | hypothetical protein | BP103_CDS_1706 | BVE_01721 | 1 |
| 92 | BP5_CDS_0111 | shell | hypothetical protein | BP5_CDS_0111 | BVE_00116 | 1 | 92 | BP103_CDS_1762 | persistent | hypothetical protein | BP103_CDS_1762 | BVE_01777 | 1 |
| 93 | BP5_CDS_0178 | shell | hypothetical protein | BP5_CDS_0178 | BVE_00183 | 1 | 93 | BP103_CDS_1773 | persistent | hypothetical protein | BP103_CDS_1773 | BVE_01788 | 1 |
| 94 | BP5_CDS_0179 | shell | hypothetical protein | BP5_CDS_0179 | BVE_00184 | 1 | 94 | BP103_CDS_1805 | persistent | hypothetical protein | BP103_CDS_1805 | BVE_01820 | 1 |
| 95 | BP5_CDS_0180 | shell | hypothetical protein | BP5_CDS_0180 | BVE_00185 | 1 | 95 | BP103_CDS_1806 | persistent | hypothetical protein | BP103_CDS_1806 | BVE_01821 | 1 |
| 96 | BP5_CDS_0182 | persistent | hypothetical protein | BP5_CDS_0182 | BVE_00187 | 1 | 96 | BP103_CDS_1810 | persistent | hypothetical protein | BP103_CDS_1810 | BVE_01825 | 1 |
| 97 | BP5_CDS_0190 | persistent | hypothetical protein | BP5_CDS_0190 | BVE_00195 | 1 | 97 | BP103_CDS_1852 | persistent | hypothetical protein | BP103_CDS_1852 | BVE_01867 | 1 |
| 98 | BP5_CDS_0274 | shell | hypothetical protein | BP5_CDS_0274 | BVE_00279 | 1 | 98 | BP103_CDS_1853 | persistent | hypothetical protein | BP103_CDS_1853 | BVE_01868 | 1 |
| 99 | BP5_CDS_0275 | shell | hypothetical protein | BP5_CDS_0275 | BVE_00280 | 1 | 99 | BP103_CDS_1913 | persistent | hypothetical protein | BP103_CDS_1913 | BVE_01932 | 1 |
| 100 | BP5_CDS_0276 | shell | hypothetical protein | BP5_CDS_0276 | BVE_00281 | 1 | 100 | BP103_CDS_1916 | persistent | hypothetical protein | BP103_CDS_1916 | BVE_01935 | 1 |
| 101 | BP5_CDS_0353 | shell | hypothetical protein | BP5_CDS_0353 | BVE_00358 | 1 | 101 | BP103_CDS_1917 | persistent | hypothetical protein | BP103_CDS_1917 | BVE_01936 | 1 |
| 102 | BP5_CDS_0466 | persistent | hypothetical protein | BP5_CDS_0466 | BVE_00471 | 1 | 102 | BP103_CDS_1960 | persistent | hypothetical protein | BP103_CDS_1960 | BVE_01979 | 1 |
| 103 | BP5_CDS_0483 | persistent | hypothetical protein | BP5_CDS_0483 | BVE_00488 | 1 | 103 | BP103_CDS_1961 | persistent | hypothetical protein | BP103_CDS_1961 | BVE_01980 | 1 |
| 104 | BP5_CDS_0616 | persistent | hypothetical protein | BP5_CDS_0616 | BVE_00621 | 1 | 104 | BP103_CDS_1964 | persistent | hypothetical protein | BP103_CDS_1964 | BVE_01983 | 1 |
| 105 | BP5_CDS_0626 | persistent | hypothetical protein | BP5_CDS_0626 | BVE_00632 | 1 | 105 | BP103_CDS_1965 | persistent | hypothetical protein | BP103_CDS_1965 | BVE_01984 | 1 |
| 106 | BP5_CDS_0662 | persistent | hypothetical protein | BP5_CDS_0662 | BVE_00668 | 1 | 106 | BP103_CDS_1966 | persistent | hypothetical protein | BP103_CDS_1966 | BVE_01985 | 1 |
| 107 | BP5_CDS_0726 | persistent | hypothetical protein | BP5_CDS_0726 | BVE_00733 | 1 | 107 | BP103_CDS_1967 | persistent | hypothetical protein | BP103_CDS_1967 | BVE_01986 | 1 |
| 108 | BP5_CDS_0839 | shell | hypothetical protein | BP5_CDS_0839 | BVE_00846 | 1 | 108 | BP103_CDS_1969 | persistent | hypothetical protein | BP103_CDS_1969 | BVE_01988 | 1 |
| 109 | BP5_CDS_0840 | shell | hypothetical protein | BP5_CDS_0840 | BVE_00847 | 1 | 109 | BP103_CDS_1970 | persistent | hypothetical protein | BP103_CDS_1970 | BVE_01989 | 1 |
| 110 | BP5_CDS_0841 | shell | hypothetical protein | BP5_CDS_0841 | BVE_00848 | 1 | 110 | BP103_CDS_1971 | persistent | hypothetical protein | BP103_CDS_1971 | BVE_01990 | 1 |
| 111 | BP5_CDS_0868 | shell | hypothetical protein | BP5_CDS_0868 | BVE_00875 | 1 | 111 | BP103_CDS_1972 | persistent | hypothetical protein | BP103_CDS_1972 | BVE_01991 | 1 |
| 112 | BP5_CDS_0870 | shell | hypothetical protein | BP5_CDS_0870 | BVE_00877 | 1 | 112 | BP103_CDS_1973 | persistent | hypothetical protein | BP103_CDS_1973 | BVE_01992 | 1 |
| 113 | BP5_CDS_0871 | shell | hypothetical protein | BP5_CDS_0871 | BVE_00878 | 1 | 113 | BP103_CDS_1974 | persistent | hypothetical protein | BP103_CDS_1974 | BVE_01993 | 1 |
| 114 | BP5_CDS_0872 | shell | hypothetical protein | BP5_CDS_0872 | BVE_00879 | 1 | 114 | BP103_CDS_1975 | persistent | hypothetical protein | BP103_CDS_1975 | BVE_01994 | 1 |
| 115 | BP5_CDS_0873 | shell | hypothetical protein | BP5_CDS_0873 | BVE_00880 | 1 | 115 | BP103_CDS_1976 | persistent | hypothetical protein | BP103_CDS_1976 | BVE_01995 | 1 |
| 116 | BP5_CDS_0874 | shell | hypothetical protein | BP5_CDS_0874 | BVE_00881 | 1 | 116 | BP103_CDS_1977 | persistent | hypothetical protein | BP103_CDS_1977 | BVE_01996 | 1 |
| 117 | BP5_CDS_0875 | shell | hypothetical protein | BP5_CDS_0875 | BVE_00882 | 1 | 117 | BP103_CDS_1978 | persistent | hypothetical protein | BP103_CDS_1978 | BVE_01997 | 1 |
| 118 | BP5_CDS_0876 | shell | hypothetical protein | BP5_CDS_0876 | BVE_00883 | 1 | 118 | BP103_CDS_1979 | persistent | hypothetical protein | BP103_CDS_1979 | BVE_01998 | 1 |
| 119 | BP5_CDS_0877 | shell | hypothetical protein | BP5_CDS_0877 | BVE_00884 | 1 | 119 | BP103_CDS_1980 | persistent | hypothetical protein | BP103_CDS_1980 | BVE_01999 | 1 |
| 120 | BP5_CDS_0878 | shell | hypothetical protein | BP5_CDS_0878 | BVE_00885 | 1 | 120 | BP103_CDS_1981 | persistent | hypothetical protein | BP103_CDS_1981 | BVE_02000 | 1 |
| 121 | BP5_CDS_0879 | shell | hypothetical protein | BP5_CDS_0879 | BVE_00886 | 1 | 121 | BP103_CDS_1982 | persistent | hypothetical protein | BP103_CDS_1982 | BVE_02001 | 1 |
| 122 | BP5_CDS_0880 | shell | hypothetical protein | BP5_CDS_0880 | BVE_00887 | 1 | 122 | BP103_CDS_1983 | persistent | hypothetical protein | BP103_CDS_1983 | BVE_02002 | 1 |
| 123 | BP5_CDS_0881 | shell | hypothetical protein | BP5_CDS_0881 | BVE_00888 | 1 | 123 | BP103_CDS_1984 | persistent | hypothetical protein | BP103_CDS_1984 | BVE_02003 | 1 |
| 124 | BP5_CDS_0882 | shell | hypothetical protein | BP5_CDS_0882 | BVE_00889 | 1 | 124 | BP103_CDS_1985 | persistent | hypothetical protein | BP103_CDS_1985 | BVE_02004 | 1 |
| 125 | BP5_CDS_0883 | shell | hypothetical protein | BP5_CDS_0883 | BVE_00890 | 1 | 125 | BP103_CDS_1986 | persistent | hypothetical protein | BP103_CDS_1986 | BVE_02005 | 1 |
| 126 | BP5_CDS_0884 | shell | hypothetical protein | BP5_CDS_0884 | BVE_00891 | 1 | 126 | BP103_CDS_1987 | persistent | hypothetical protein | BP103_CDS_1987 | BVE_02006 | 1 |
| 127 | BP5_CDS_0885 | shell | hypothetical protein | BP5_CDS_0885 | BVE_00892 | 1 | 127 | BP103_CDS_1988 | persistent | hypothetical protein | BP103_CDS_1988 | BVE_02007 | 1 |
| 128 | BP5_CDS_0886 | shell | hypothetical protein | BP5_CDS_0886 | BVE_00893 | 1 | 128 | BP103_CDS_1989 | persistent | hypothetical protein | BP103_CDS_1989 | BVE_02008 | 1 |
| 129 | BP5_CDS_0887 | shell | hypothetical protein | BP5_CDS_0887 | BVE_00894 | 1 | 129 | BP103_CDS_1990 | persistent | hypothetical protein | BP103_CDS_1990 | BVE_02009 | 1 |
| 130 | BP5_CDS_0888 | shell | hypothetical protein | BP5_CDS_0888 | BVE_00895 | 1 | 130 | BP103_CDS_1992 | persistent | hypothetical protein | BP103_CDS_1992 | BVE_02011 | 1 |
| 131 | BP5_CDS_0889 | shell | hypothetical protein | BP5_CDS_0889 | BVE_00896 | 1 | 131 | BP103_CDS_1993 | persistent | hypothetical protein | BP103_CDS_1993 | BVE_02012 | 1 |
| 132 | BP5_CDS_0936 | persistent | hypothetical protein | BP5_CDS_0936 | BVE_00943 | 1 | 132 | BP103_CDS_1994 | persistent | hypothetical protein | BP103_CDS_1994 | BVE_02013 | 1 |
| 133 | BP5_CDS_0997 | shell | hypothetical protein | BP5_CDS_0997 | BVE_01005 | 1 | 133 | BP103_CDS_1996 | persistent | hypothetical protein | BP103_CDS_1996 | BVE_02015 | 1 |
| 134 | BP5_CDS_0998 | shell | hypothetical protein | BP5_CDS_0998 | BVE_01006 | 1 | 134 | BP103_CDS_1997 | persistent | hypothetical protein | BP103_CDS_1997 | BVE_02016 | 1 |
| 135 | BP5_CDS_0999 | shell | hypothetical protein | BP5_CDS_0999 | BVE_01007 | 1 | 135 | BP103_CDS_1998 | persistent | hypothetical protein | BP103_CDS_1998 | BVE_02017 | 1 |
| 136 | BP5_CDS_1001 | shell | hypothetical protein | BP5_CDS_1001 | BVE_01009 | 1 | 136 | BP103_CDS_1999 | persistent | hypothetical protein | BP103_CDS_1999 | BVE_02018 | 1 |
| 137 | BP5_CDS_1002 | shell | hypothetical protein | BP5_CDS_1002 | BVE_01010 | 1 | 137 | BP103_CDS_2000 | persistent | hypothetical protein | BP103_CDS_2000 | BVE_02019 | 1 |
| 138 | BP5_CDS_1003 | shell | hypothetical protein | BP5_CDS_1003 | BVE_01011 | 1 | 138 | BP103_CDS_2001 | persistent | hypothetical protein | BP103_CDS_2001 | BVE_02020 | 1 |
| 139 | BP5_CDS_1004 | shell | hypothetical protein | BP5_CDS_1004 | BVE_01012 | 1 | 139 | BP103_CDS_2002 | persistent | hypothetical protein | BP103_CDS_2002 | BVE_02021 | 1 |
| 140 | BP5_CDS_1006 | shell | hypothetical protein | BP5_CDS_1006 | BVE_01014 | 1 | 140 | BP103_CDS_2003 | persistent | hypothetical protein | BP103_CDS_2003 | BVE_02022 | 1 |
| 141 | BP5_CDS_1007 | shell | hypothetical protein | BP5_CDS_1007 | BVE_01015 | 1 | 141 | BP103_CDS_2004 | persistent | hypothetical protein | BP103_CDS_2004 | BVE_02023 | 1 |
| 142 | BP5_CDS_1008 | shell | hypothetical protein | BP5_CDS_1008 | BVE_01016 | 1 | 142 | BP103_CDS_2005 | persistent | hypothetical protein | BP103_CDS_2005 | BVE_02024 | 1 |
| 143 | BP5_CDS_1009 | shell | hypothetical protein | BP5_CDS_1009 | BVE_01017 | 1 | 143 | BP103_CDS_2006 | persistent | hypothetical protein | BP103_CDS_2006 | BVE_02025 | 1 |
| 144 | BP5_CDS_1010 | shell | hypothetical protein | BP5_CDS_1010 | BVE_01018 | 1 | 144 | BP103_CDS_2007 | persistent | hypothetical protein | BP103_CDS_2007 | BVE_02026 | 1 |
| 145 | BP5_CDS_1012 | shell | hypothetical protein | BP5_CDS_1012 | BVE_01020 | 1 | 145 | BP103_CDS_2008 | persistent | hypothetical protein | BP103_CDS_2008 | BVE_02027 | 1 |
| 146 | BP5_CDS_1013 | shell | hypothetical protein | BP5_CDS_1013 | BVE_01021 | 1 | 146 | BP103_CDS_2010 | persistent | hypothetical protein | BP103_CDS_2010 | BVE_02029 | 1 |
| 147 | BP5_CDS_1014 | shell | hypothetical protein | BP5_CDS_1014 | BVE_01022 | 1 | 147 | BP103_CDS_2011 | persistent | hypothetical protein | BP103_CDS_2011 | BVE_02030 | 1 |
| 148 | BP5_CDS_1015 | shell | hypothetical protein | BP5_CDS_1015 | BVE_01023 | 1 | 148 | BP103_CDS_2012 | persistent | hypothetical protein | BP103_CDS_2012 | BVE_02031 | 1 |
| 149 | BP5_CDS_1016 | shell | hypothetical protein | BP5_CDS_1016 | BVE_01024 | 1 | 149 | BP103_CDS_2035 | persistent | hypothetical protein | BP103_CDS_2035 | BVE_02055 | 1 |
| 150 | BP5_CDS_1018 | shell | hypothetical protein | BP5_CDS_1018 | BVE_01026 | 1 | 150 | BP103_CDS_2143 | persistent | hypothetical protein | BP103_CDS_2143 | BVE_02163 | 1 |
| 151 | BP5_CDS_1019 | shell | hypothetical protein | BP5_CDS_1019 | BVE_01027 | 1 | 151 | BP103_CDS_2568 | persistent | hypothetical protein | BP103_CDS_2568 | BVE_02589 | 1 |
| 152 | BP5_CDS_1020 | shell | hypothetical protein | BP5_CDS_1020 | BVE_01028 | 1 | 152 | BP103_CDS_2569 | persistent | hypothetical protein | BP103_CDS_2569 | BVE_02590 | 1 |
| 153 | BP5_CDS_1021 | shell | hypothetical protein | BP5_CDS_1021 | BVE_01029 | 1 | 153 | BP103_CDS_2572 | persistent | hypothetical protein | BP103_CDS_2572 | BVE_02593 | 1 |
| 154 | BP5_CDS_1022 | shell | hypothetical protein | BP5_CDS_1022 | BVE_01030 | 1 | 154 | BP103_CDS_2574 | persistent | hypothetical protein | BP103_CDS_2574 | BVE_02595 | 1 |
| 155 | BP5_CDS_1023 | shell | hypothetical protein | BP5_CDS_1023 | BVE_01031 | 1 | 155 | BP103_CDS_2577 | persistent | hypothetical protein | BP103_CDS_2577 | BVE_02598 | 1 |
| 156 | BP5_CDS_1024 | shell | hypothetical protein | BP5_CDS_1024 | BVE_01032 | 1 | 156 | BP103_CDS_2582 | persistent | hypothetical protein | BP103_CDS_2582 | BVE_02603 | 1 |
| 157 | BP5_CDS_1025 | shell | hypothetical protein | BP5_CDS_1025 | BVE_01033 | 1 | 157 | BP103_CDS_2835 | persistent | hypothetical protein | BP103_CDS_2835 | BVE_02858 | 1 |
| 158 | BP5_CDS_1026 | shell | hypothetical protein | BP5_CDS_1026 | BVE_01034 | 1 | 158 | BP103_CDS_2836 | persistent | hypothetical protein | BP103_CDS_2836 | BVE_02859 | 1 |
| 159 | BP5_CDS_1027 | shell | hypothetical protein | BP5_CDS_1027 | BVE_01035 | 1 | 159 | BP103_CDS_2837 | persistent | hypothetical protein | BP103_CDS_2837 | BVE_02860 | 1 |
| 160 | BP5_CDS_1029 | shell | hypothetical protein | BP5_CDS_1029 | BVE_01037 | 1 | 160 | BP103_CDS_2838 | persistent | hypothetical protein | BP103_CDS_2838 | BVE_02861 | 1 |
| 161 | BP5_CDS_1030 | shell | hypothetical protein | BP5_CDS_1030 | BVE_01038 | 1 | 161 | BP103_CDS_2839 | persistent | hypothetical protein | BP103_CDS_2839 | BVE_02862 | 1 |
| 162 | BP5_CDS_1031 | shell | hypothetical protein | BP5_CDS_1031 | BVE_01039 | 1 | 162 | BP103_CDS_3003 | persistent | hypothetical protein | BP103_CDS_3003 | BVE_03034 | 1 |
| 163 | BP5_CDS_1032 | shell | hypothetical protein | BP5_CDS_1032 | BVE_01040 | 1 | 163 | BP103_CDS_3006 | persistent | hypothetical protein | BP103_CDS_3006 | BVE_03037 | 1 |
| 164 | BP5_CDS_1033 | shell | hypothetical protein | BP5_CDS_1033 | BVE_01041 | 1 | 164 | BP103_CDS_3007 | persistent | hypothetical protein | BP103_CDS_3007 | BVE_03038 | 1 |
| 165 | BP5_CDS_1034 | shell | hypothetical protein | BP5_CDS_1034 | BVE_01042 | 1 | 165 | BP103_CDS_3010 | persistent | hypothetical protein | BP103_CDS_3010 | BVE_03041 | 1 |
| 166 | BP5_CDS_1035 | shell | hypothetical protein | BP5_CDS_1035 | BVE_01043 | 1 | 166 | BP103_CDS_3030 | persistent | hypothetical protein | BP103_CDS_3030 | BVE_03061 | 1 |
| 167 | BP5_CDS_1036 | shell | hypothetical protein | BP5_CDS_1036 | BVE_01044 | 1 | 167 | BP103_CDS_3031 | persistent | hypothetical protein | BP103_CDS_3031 | BVE_03062 | 1 |
| 168 | BP5_CDS_1037 | shell | hypothetical protein | BP5_CDS_1037 | BVE_01045 | 1 | 168 | BP103_CDS_3032 | persistent | hypothetical protein | BP103_CDS_3032 | BVE_03063 | 1 |
| 169 | BP5_CDS_1039 | shell | hypothetical protein | BP5_CDS_1039 | BVE_01047 | 1 | 169 | BP103_CDS_3035 | persistent | hypothetical protein | BP103_CDS_3035 | BVE_03066 | 1 |
| 170 | BP5_CDS_1040 | shell | hypothetical protein | BP5_CDS_1040 | BVE_01048 | 1 | 170 | BP103_CDS_3072 | persistent | hypothetical protein | BP103_CDS_3072 | BVE_03110 | 1 |
| 171 | BP5_CDS_1042 | shell | hypothetical protein | BP5_CDS_1042 | BVE_01050 | 1 | 171 | BP103_CDS_3269 | persistent | hypothetical protein | BP103_CDS_3269 | BVE_03307 | 1 |
| 172 | BP5_CDS_1043 | shell | hypothetical protein | BP5_CDS_1043 | BVE_01051 | 1 | 172 | BP103_CDS_3274 | persistent | hypothetical protein | BP103_CDS_3274 | BVE_03312 | 1 |
| 173 | BP5_CDS_1044 | shell | hypothetical protein | BP5_CDS_1044 | BVE_01052 | 1 | 173 | BP103_CDS_3275 | persistent | hypothetical protein | BP103_CDS_3275 | BVE_03313 | 1 |
| 174 | BP5_CDS_1045 | shell | hypothetical protein | BP5_CDS_1045 | BVE_01053 | 1 | 174 | BP103_CDS_3276 | persistent | hypothetical protein | BP103_CDS_3276 | BVE_03314 | 1 |
| 175 | BP5_CDS_1046 | shell | hypothetical protein | BP5_CDS_1046 | BVE_01054 | 1 | 175 | BP103_CDS_3277 | persistent | hypothetical protein | BP103_CDS_3277 | BVE_03315 | 1 |
| 176 | BP5_CDS_1047 | shell | hypothetical protein | BP5_CDS_1047 | BVE_01055 | 1 | 176 | BP103_CDS_3300 | persistent | hypothetical protein | BP103_CDS_3300 | BVE_03338 | 1 |
| 177 | BP5_CDS_1050 | shell | hypothetical protein | BP5_CDS_1050 | BVE_01058 | 1 | 177 | BP103_CDS_3305 | persistent | hypothetical protein | BP103_CDS_3305 | BVE_03343 | 1 |
| 178 | BP5_CDS_1052 | shell | hypothetical protein | BP5_CDS_1052 | BVE_01060 | 1 | 178 | BP103_CDS_3306 | persistent | hypothetical protein | BP103_CDS_3306 | BVE_03344 | 1 |
| 179 | BP5_CDS_1053 | shell | hypothetical protein | BP5_CDS_1053 | BVE_01061 | 1 | 179 | BP103_CDS_3347 | persistent | hypothetical protein | BP103_CDS_3347 | BVE_03392 | 1 |
| 180 | BP5_CDS_1055 | shell | hypothetical protein | BP5_CDS_1055 | BVE_01063 | 1 | 180 | BP103_CDS_3349 | persistent | hypothetical protein | BP103_CDS_3349 | BVE_03394 | 1 |
| 181 | BP5_CDS_1071 | shell | hypothetical protein | BP5_CDS_1071 | BVE_01079 | 1 | 181 | BP103_CDS_3351 | persistent | hypothetical protein | BP103_CDS_3351 | BVE_03396 | 1 |
| 182 | BP5_CDS_1072 | shell | hypothetical protein | BP5_CDS_1072 | BVE_01080 | 1 | 182 | BP103_CDS_3414 | persistent | hypothetical protein | BP103_CDS_3414 | BVE_03459 | 1 |
| 183 | BP5_CDS_1073 | shell | hypothetical protein | BP5_CDS_1073 | BVE_01081 | 1 | 183 | BP103_CDS_3415 | persistent | hypothetical protein | BP103_CDS_3415 | BVE_03460 | 1 |
| 184 | BP5_CDS_1075 | shell | hypothetical protein | BP5_CDS_1075 | BVE_01083 | 1 | 184 | BP103_CDS_3416 | persistent | hypothetical protein | BP103_CDS_3416 | BVE_03461 | 1 |
| 185 | BP5_CDS_1076 | shell | hypothetical protein | BP5_CDS_1076 | BVE_01084 | 1 | 185 | BP103_CDS_3417 | persistent | hypothetical protein | BP103_CDS_3417 | BVE_03462 | 1 |
| 186 | BP5_CDS_1077 | shell | hypothetical protein | BP5_CDS_1077 | BVE_01085 | 1 | 186 | BP103_CDS_3444 | persistent | hypothetical protein | BP103_CDS_3444 | BVE_03489 | 1 |
| 187 | BP5_CDS_1078 | shell | hypothetical protein | BP5_CDS_1078 | BVE_01086 | 1 | 187 | BP103_CDS_3445 | persistent | hypothetical protein | BP103_CDS_3445 | BVE_03490 | 1 |
| 188 | BP5_CDS_1079 | shell | hypothetical protein | BP5_CDS_1079 | BVE_01087 | 1 | 188 | BP103_CDS_3448 | persistent | hypothetical protein | BP103_CDS_3448 | BVE_03493 | 1 |
| 189 | BP5_CDS_1080 | shell | hypothetical protein | BP5_CDS_1080 | BVE_01088 | 1 | 189 | BP103_CDS_3473 | persistent | hypothetical protein | BP103_CDS_3473 | BVE_03518 | 1 |
| 190 | BP5_CDS_1081 | shell | hypothetical protein | BP5_CDS_1081 | BVE_01089 | 1 | 190 | BP103_CDS_3544 | persistent | hypothetical protein | BP103_CDS_3544 | BVE_03589 | 1 |
| 191 | BP5_CDS_1082 | shell | hypothetical protein | BP5_CDS_1082 | BVE_01090 | 1 | 191 | BP103_CDS_3731 | persistent | hypothetical protein | BP103_CDS_3731 | BVE_03779 | 1 |
| 192 | BP5_CDS_1083 | shell | hypothetical protein | BP5_CDS_1083 | BVE_01091 | 1 | 192 | BP103_CDS_3732 | persistent | hypothetical protein | BP103_CDS_3732 | BVE_03780 | 1 |
| 193 | BP5_CDS_1084 | shell | hypothetical protein | BP5_CDS_1084 | BVE_01092 | 1 | 193 | BP103_CDS_1804 | persistent | putative ABC transporter ATP-binding protein YxlF | BP103_CDS_1804 | BVE_01819 | 1 |
| 194 | BP5_CDS_1085 | shell | hypothetical protein | BP5_CDS_1085 | BVE_01093 | 1 | 194 | BP103_CDS_2576 | persistent | putative HTH-type transcriptional regulator | BP103_CDS_2576 | BVE_02597 | 1 |
| 195 | BP5_CDS_1086 | shell | hypothetical protein | BP5_CDS_1086 | BVE_01094 | 1 | 195 | BP103_CDS_3017 | persistent | putative N-acetyltransferase YsnE | BP103_CDS_3017 | BVE_03048 | 1 |
| 196 | BP5_CDS_1087 | shell | hypothetical protein | BP5_CDS_1087 | BVE_01095 | 1 | 196 | BP103_CDS_1603 | persistent | putative beta-barrel protein YwiB | BP103_CDS_1603 | BVE_01618 | 1 |
| 197 | BP5_CDS_1088 | shell | hypothetical protein | BP5_CDS_1088 | BVE_01096 | 1 | 197 | BP103_CDS_1920 | persistent | putative membrane protein YdfK | BP103_CDS_1920 | BVE_01939 | 1 |
| 198 | BP5_CDS_1089 | shell | hypothetical protein | BP5_CDS_1089 | BVE_01097 | 1 | 198 | BP103_CDS_3447 | persistent | putative multidrug ABC transporter permease YbhR | BP103_CDS_3447 | BVE_03492 | 1 |
| 199 | BP5_CDS_1090 | shell | hypothetical protein | BP5_CDS_1090 | BVE_01098 | 1 | 199 | BP103_CDS_1105 | persistent | putative protein YqbD | BP103_CDS_1105 | BVE_01118 | 1 |
| 200 | BP5_CDS_1293 | persistent | hypothetical protein | BP5_CDS_1293 | BVE_01301 | 1 | 200 | BP103_CDS_1604 | persistent | putative protein YwhK | BP103_CDS_1604 | BVE_01619 | 1 |
| 201 | BP5_CDS_1294 | persistent | hypothetical protein | BP5_CDS_1294 | BVE_01302 | 1 | 201 | BP103_CDS_1605 | persistent | putative protein YwhK | BP103_CDS_1605 | BVE_01620 | 1 |
| 202 | BP5_CDS_1333 | shell | hypothetical protein | BP5_CDS_1333 | BVE_01341 | 1 | 202 | BP103_CDS_3348 | persistent | putative ribonuclease YokI | BP103_CDS_3348 | BVE_03393 | 1 |
| 203 | BP5_CDS_1335 | shell | hypothetical protein | BP5_CDS_1334;  BP5_CDS_1335 | BVE_01342;  BVE_01343 | 2 | 203 | BP103_CDS_3350 | persistent | putative type I restriction enzymeP M protein | BP103_CDS_3350 | BVE_03395 | 1 |
| 204 | BP5_CDS_1337 | shell | hypothetical protein | BP5_CDS_1337 | BVE_01345 | 1 |  |  |  |  |  |  |  |
| 205 | BP5_CDS_1338 | shell | hypothetical protein | BP5_CDS_1338 | BVE_01346 | 1 |  |  |  |  |  |  |  |
| 206 | BP5_CDS_1341 | shell | hypothetical protein | BP5_CDS_1341 | BVE_01349 | 1 |  |  |  |  |  |  |  |
| 207 | BP5_CDS_1342 | shell | hypothetical protein | BP5_CDS_1342 | BVE_01350 | 1 |  |  |  |  |  |  |  |
| 208 | BP5_CDS_1385 | persistent | hypothetical protein | BP5_CDS_1385 | BVE_01393 | 1 |  |  |  |  |  |  |  |
| 209 | BP5_CDS_1391 | persistent | hypothetical protein | BP5_CDS_1391 | BVE_01400 | 1 |  |  |  |  |  |  |  |
| 210 | BP5_CDS_1443 | persistent | hypothetical protein | BP5_CDS_1443 | BVE_01452 | 1 |  |  |  |  |  |  |  |
| 211 | BP5_CDS_1514 | persistent | hypothetical protein | BP5_CDS_1514 | BVE_01523 | 1 |  |  |  |  |  |  |  |
| 212 | BP5_CDS_1542 | persistent | hypothetical protein | BP5_CDS_1542 | BVE_01551 | 1 |  |  |  |  |  |  |  |
| 213 | BP5_CDS_1549 | persistent | hypothetical protein | BP5_CDS_1549 | BVE_01558 | 1 |  |  |  |  |  |  |  |
| 214 | BP5_CDS_1690 | persistent | hypothetical protein | BP5_CDS_1690 | BVE_01700 | 1 |  |  |  |  |  |  |  |
| 215 | BP5_CDS_1895 | persistent | hypothetical protein | BP5_CDS_1895 | BVE_01915 | 1 |  |  |  |  |  |  |  |
| 216 | BP5_CDS_2295 | persistent | hypothetical protein | BP5_CDS_2295 | BVE_02323 | 1 |  |  |  |  |  |  |  |
| 217 | BP5_CDS_2357 | persistent | hypothetical protein | BP5_CDS_2357 | BVE_02385 | 1 |  |  |  |  |  |  |  |
| 218 | BP5_CDS_2386 | persistent | hypothetical protein | BP5_CDS_2386 | BVE_02414 | 1 |  |  |  |  |  |  |  |
| 219 | BP5_CDS_2406 | persistent | hypothetical protein | BP5_CDS_2406 | BVE_02434 | 1 |  |  |  |  |  |  |  |
| 220 | BP5_CDS_2407 | persistent | hypothetical protein | BP5_CDS_2407 | BVE_02435 | 1 |  |  |  |  |  |  |  |
| 221 | BP5_CDS_2629 | shell | hypothetical protein | BP5_CDS_2629 | BVE_02657 | 1 |  |  |  |  |  |  |  |
| 222 | BP5_CDS_2630 | shell | hypothetical protein | BP5_CDS_2630 | BVE_02658 | 1 |  |  |  |  |  |  |  |
| 223 | BP5_CDS_2631 | shell | hypothetical protein | BP5_CDS_2631 | BVE_02659 | 1 |  |  |  |  |  |  |  |
| 224 | BP5_CDS_2632 | shell | hypothetical protein | BP5_CDS_2632 | BVE_02660 | 1 |  |  |  |  |  |  |  |
| 225 | BP5_CDS_2633 | shell | hypothetical protein | BP5_CDS_2633 | BVE_02661 | 1 |  |  |  |  |  |  |  |
| 226 | BP5_CDS_2634 | shell | hypothetical protein | BP5_CDS_2634 | BVE_02662 | 1 |  |  |  |  |  |  |  |
| 227 | BP5_CDS_2635 | shell | hypothetical protein | BP5_CDS_2635 | BVE_02663 | 1 |  |  |  |  |  |  |  |
| 228 | BP5_CDS_2636 | shell | hypothetical protein | BP5_CDS_2636 | BVE_02664 | 1 |  |  |  |  |  |  |  |
| 229 | BP5_CDS_2637 | shell | hypothetical protein | BP5_CDS_2637 | BVE_02665 | 1 |  |  |  |  |  |  |  |
| 230 | BP5_CDS_2638 | shell | hypothetical protein | BP5_CDS_2638 | BVE_02666 | 1 |  |  |  |  |  |  |  |
| 231 | BP5_CDS_2639 | shell | hypothetical protein | BP5_CDS_2639 | BVE_02667 | 1 |  |  |  |  |  |  |  |
| 232 | BP5_CDS_2640 | shell | hypothetical protein | BP5_CDS_2640 | BVE_02668 | 1 |  |  |  |  |  |  |  |
| 233 | BP5_CDS_2641 | shell | hypothetical protein | BP5_CDS_2641 | BVE_02669 | 1 |  |  |  |  |  |  |  |
| 234 | BP5_CDS_2642 | shell | hypothetical protein | BP5_CDS_2642 | BVE_02670 | 1 |  |  |  |  |  |  |  |
| 235 | BP5_CDS_2643 | shell | hypothetical protein | BP5_CDS_2643 | BVE_02671 | 1 |  |  |  |  |  |  |  |
| 236 | BP5_CDS_2644 | shell | hypothetical protein | BP5_CDS_2644 | BVE_02672 | 1 |  |  |  |  |  |  |  |
| 237 | BP5_CDS_2645 | shell | hypothetical protein | BP5_CDS_2645 | BVE_02673 | 1 |  |  |  |  |  |  |  |
| 238 | BP5_CDS_2646 | shell | hypothetical protein | BP5_CDS_2646 | BVE_02674 | 1 |  |  |  |  |  |  |  |
| 239 | BP5_CDS_2647 | shell | hypothetical protein | BP5_CDS_2647 | BVE_02675 | 1 |  |  |  |  |  |  |  |
| 240 | BP5_CDS_2648 | shell | hypothetical protein | BP5_CDS_2648 | BVE_02676 | 1 |  |  |  |  |  |  |  |
| 241 | BP5_CDS_2649 | shell | hypothetical protein | BP5_CDS_2649 | BVE_02677 | 1 |  |  |  |  |  |  |  |
| 242 | BP5_CDS_2650 | shell | hypothetical protein | BP5_CDS_2650 | BVE_02678 | 1 |  |  |  |  |  |  |  |
| 243 | BP5_CDS_2651 | shell | hypothetical protein | BP5_CDS_2651 | BVE_02679 | 1 |  |  |  |  |  |  |  |
| 244 | BP5_CDS_2652 | shell | hypothetical protein | BP5_CDS_2652 | BVE_02680 | 1 |  |  |  |  |  |  |  |
| 245 | BP5_CDS_2653 | shell | hypothetical protein | BP5_CDS_2653 | BVE_02681 | 1 |  |  |  |  |  |  |  |
| 246 | BP5_CDS_2654 | shell | hypothetical protein | BP5_CDS_2654 | BVE_02682 | 1 |  |  |  |  |  |  |  |
| 247 | BP5_CDS_2656 | shell | hypothetical protein | BP5_CDS_2656 | BVE_02684 | 1 |  |  |  |  |  |  |  |
| 248 | BP5_CDS_2657 | persistent | hypothetical protein | BP5_CDS_2657 | BVE_02685 | 1 |  |  |  |  |  |  |  |
| 249 | BP5_CDS_2683 | persistent | hypothetical protein | BP5_CDS_2683 | BVE_02712 | 1 |  |  |  |  |  |  |  |
| 250 | BP5_CDS_2779 | persistent | hypothetical protein | BP5_CDS_2779 | BVE_02808 | 1 |  |  |  |  |  |  |  |
| 251 | BP5_CDS_2781 | shell | hypothetical protein | BP5_CDS_2781 | BVE_02810 | 1 |  |  |  |  |  |  |  |
| 252 | BP5_CDS_2782 | shell | hypothetical protein | BP5_CDS_2782 | BVE_02811 | 1 |  |  |  |  |  |  |  |
| 253 | BP5_CDS_2783 | shell | hypothetical protein | BP5_CDS_2783 | BVE_02812 | 1 |  |  |  |  |  |  |  |
| 254 | BP5_CDS_2784 | shell | hypothetical protein | BP5_CDS_2784 | BVE_02813 | 1 |  |  |  |  |  |  |  |
| 255 | BP5_CDS_2785 | shell | hypothetical protein | BP5_CDS_2785 | BVE_02814 | 1 |  |  |  |  |  |  |  |
| 256 | BP5_CDS_2790 | persistent | hypothetical protein | BP5_CDS_2790 | BVE_02819 | 1 |  |  |  |  |  |  |  |
| 257 | BP5_CDS_2794 | shell | hypothetical protein | BP5_CDS_2794 | BVE_02823 | 1 |  |  |  |  |  |  |  |
| 258 | BP5_CDS_2795 | shell | hypothetical protein | BP5_CDS_2795 | BVE_02824 | 1 |  |  |  |  |  |  |  |
| 259 | BP5_CDS_2796 | shell | hypothetical protein | BP5_CDS_2796 | BVE_02825 | 1 |  |  |  |  |  |  |  |
| 260 | BP5_CDS_2799 | persistent | hypothetical protein | BP5_CDS_2799 | BVE_02828 | 1 |  |  |  |  |  |  |  |
| 261 | BP5_CDS_2800 | persistent | hypothetical protein | BP5_CDS_2800 | BVE_02829 | 1 |  |  |  |  |  |  |  |
| 262 | BP5_CDS_2848 | shell | hypothetical protein | BP5_CDS_2848 | BVE_02877 | 1 |  |  |  |  |  |  |  |
| 263 | BP5_CDS_2849 | shell | hypothetical protein | BP5_CDS_2849 | BVE_02878 | 1 |  |  |  |  |  |  |  |
| 264 | BP5_CDS_2850 | shell | hypothetical protein | BP5_CDS_2850 | BVE_02879 | 1 |  |  |  |  |  |  |  |
| 265 | BP5_CDS_2851 | shell | hypothetical protein | BP5_CDS_2851 | BVE_02880 | 1 |  |  |  |  |  |  |  |
| 266 | BP5_CDS_2852 | shell | hypothetical protein | BP5_CDS_2852 | BVE_02881 | 1 |  |  |  |  |  |  |  |
| 267 | BP5_CDS_2853 | shell | hypothetical protein | BP5_CDS_2853 | BVE_02882 | 1 |  |  |  |  |  |  |  |
| 268 | BP5_CDS_2854 | shell | hypothetical protein | BP5_CDS_2854 | BVE_02883 | 1 |  |  |  |  |  |  |  |
| 269 | BP5_CDS_2855 | shell | hypothetical protein | BP5_CDS_2855 | BVE_02884 | 1 |  |  |  |  |  |  |  |
| 270 | BP5_CDS_2856 | shell | hypothetical protein | BP5_CDS_2856 | BVE_02885 | 1 |  |  |  |  |  |  |  |
| 271 | BP5_CDS_2857 | shell | hypothetical protein | BP5_CDS_2857 | BVE_02886 | 1 |  |  |  |  |  |  |  |
| 272 | BP5_CDS_2858 | shell | hypothetical protein | BP5_CDS_2858 | BVE_02887 | 1 |  |  |  |  |  |  |  |
| 273 | BP5_CDS_2859 | shell | hypothetical protein | BP5_CDS_2859 | BVE_02888 | 1 |  |  |  |  |  |  |  |
| 274 | BP5_CDS_2860 | shell | hypothetical protein | BP5_CDS_2860 | BVE_02889 | 1 |  |  |  |  |  |  |  |
| 275 | BP5_CDS_2863 | shell | hypothetical protein | BP5_CDS_2863 | BVE_02892 | 1 |  |  |  |  |  |  |  |
| 276 | BP5_CDS_2864 | shell | hypothetical protein | BP5_CDS_2864 | BVE_02893 | 1 |  |  |  |  |  |  |  |
| 277 | BP5_CDS_2865 | shell | hypothetical protein | BP5_CDS_2865 | BVE_02894 | 1 |  |  |  |  |  |  |  |
| 278 | BP5_CDS_2866 | shell | hypothetical protein | BP5_CDS_2866 | BVE_02895 | 1 |  |  |  |  |  |  |  |
| 279 | BP5_CDS_2867 | shell | hypothetical protein | BP5_CDS_2867 | BVE_02896 | 1 |  |  |  |  |  |  |  |
| 280 | BP5_CDS_2868 | shell | hypothetical protein | BP5_CDS_2868 | BVE_02897 | 1 |  |  |  |  |  |  |  |
| 281 | BP5_CDS_2871 | shell | hypothetical protein | BP5_CDS_2871 | BVE_02900 | 1 |  |  |  |  |  |  |  |
| 282 | BP5_CDS_2872 | shell | hypothetical protein | BP5_CDS_2872 | BVE_02901 | 1 |  |  |  |  |  |  |  |
| 283 | BP5_CDS_2873 | shell | hypothetical protein | BP5_CDS_2873 | BVE_02902 | 1 |  |  |  |  |  |  |  |
| 284 | BP5_CDS_2875 | shell | hypothetical protein | BP5_CDS_2875 | BVE_02904 | 1 |  |  |  |  |  |  |  |
| 285 | BP5_CDS_2876 | shell | hypothetical protein | BP5_CDS_2876 | BVE_02905 | 1 |  |  |  |  |  |  |  |
| 286 | BP5_CDS_2879 | persistent | hypothetical protein | BP5_CDS_2879 | BVE_02908 | 1 |  |  |  |  |  |  |  |
| 287 | BP5_CDS_2883 | shell | hypothetical protein | BP5_CDS_2883 | BVE_02912 | 1 |  |  |  |  |  |  |  |
| 288 | BP5_CDS_2884 | shell | hypothetical protein | BP5_CDS_2884 | BVE_02913 | 1 |  |  |  |  |  |  |  |
| 289 | BP5_CDS_2885 | shell | hypothetical protein | BP5_CDS_2885 | BVE_02914 | 1 |  |  |  |  |  |  |  |
| 290 | BP5_CDS_2886 | shell | hypothetical protein | BP5_CDS_2886 | BVE_02915 | 1 |  |  |  |  |  |  |  |
| 291 | BP5_CDS_2887 | shell | hypothetical protein | BP5_CDS_2887 | BVE_02916 | 1 |  |  |  |  |  |  |  |
| 292 | BP5_CDS_2888 | shell | hypothetical protein | BP5_CDS_2888 | BVE_02917 | 1 |  |  |  |  |  |  |  |
| 293 | BP5_CDS_2889 | shell | hypothetical protein | BP5_CDS_2889 | BVE_02918 | 1 |  |  |  |  |  |  |  |
| 294 | BP5_CDS_2890 | shell | hypothetical protein | BP5_CDS_2890 | BVE_02919 | 1 |  |  |  |  |  |  |  |
| 295 | BP5_CDS_2891 | shell | hypothetical protein | BP5_CDS_2891 | BVE_02920 | 1 |  |  |  |  |  |  |  |
| 296 | BP5_CDS_2892 | shell | hypothetical protein | BP5_CDS_2892 | BVE_02921 | 1 |  |  |  |  |  |  |  |
| 297 | BP5_CDS_2893 | shell | hypothetical protein | BP5_CDS_2893 | BVE_02922 | 1 |  |  |  |  |  |  |  |
| 298 | BP5_CDS_2896 | shell | hypothetical protein | BP5_CDS_2896 | BVE_02925 | 1 |  |  |  |  |  |  |  |
| 299 | BP5_CDS_2901 | shell | hypothetical protein | BP5_CDS_2901 | BVE_02930 | 1 |  |  |  |  |  |  |  |
| 300 | BP5_CDS_2902 | shell | hypothetical protein | BP5_CDS_2902 | BVE_02931 | 1 |  |  |  |  |  |  |  |
| 301 | BP5_CDS_2903 | shell | hypothetical protein | BP5_CDS_2903 | BVE_02932 | 1 |  |  |  |  |  |  |  |
| 302 | BP5_CDS_2904 | shell | hypothetical protein | BP5_CDS_2904 | BVE_02933 | 1 |  |  |  |  |  |  |  |
| 303 | BP5_CDS_2905 | shell | hypothetical protein | BP5_CDS_2905 | BVE_02934 | 1 |  |  |  |  |  |  |  |
| 304 | BP5_CDS_2906 | shell | hypothetical protein | BP5_CDS_2906 | BVE_02935 | 1 |  |  |  |  |  |  |  |
| 305 | BP5_CDS_2909 | shell | hypothetical protein | BP5_CDS_2909 | BVE_02938 | 1 |  |  |  |  |  |  |  |
| 306 | BP5_CDS_2910 | shell | hypothetical protein | BP5_CDS_2910 | BVE_02939 | 1 |  |  |  |  |  |  |  |
| 307 | BP5_CDS_2911 | shell | hypothetical protein | BP5_CDS_2911 | BVE_02940 | 1 |  |  |  |  |  |  |  |
| 308 | BP5_CDS_2912 | shell | hypothetical protein | BP5_CDS_2912 | BVE_02941 | 1 |  |  |  |  |  |  |  |
| 309 | BP5_CDS_2913 | shell | hypothetical protein | BP5_CDS_2913 | BVE_02942 | 1 |  |  |  |  |  |  |  |
| 310 | BP5_CDS_2916 | shell | hypothetical protein | BP5_CDS_2916 | BVE_02945 | 1 |  |  |  |  |  |  |  |
| 311 | BP5_CDS_2918 | shell | hypothetical protein | BP5_CDS_2918 | BVE_02947 | 1 |  |  |  |  |  |  |  |
| 312 | BP5_CDS_2919 | shell | hypothetical protein | BP5_CDS_2919 | BVE_02948 | 1 |  |  |  |  |  |  |  |
| 313 | BP5_CDS_2920 | shell | hypothetical protein | BP5_CDS_2920 | BVE_02949 | 1 |  |  |  |  |  |  |  |
| 314 | BP5_CDS_2921 | shell | hypothetical protein | BP5_CDS_2921 | BVE_02950 | 1 |  |  |  |  |  |  |  |
| 315 | BP5_CDS_2922 | shell | hypothetical protein | BP5_CDS_2922 | BVE_02951 | 1 |  |  |  |  |  |  |  |
| 316 | BP5_CDS_2923 | shell | hypothetical protein | BP5_CDS_2923 | BVE_02952 | 1 |  |  |  |  |  |  |  |
| 317 | BP5_CDS_2924 | shell | hypothetical protein | BP5_CDS_2924 | BVE_02953 | 1 |  |  |  |  |  |  |  |
| 318 | BP5_CDS_2925 | shell | hypothetical protein | BP5_CDS_2925 | BVE_02954 | 1 |  |  |  |  |  |  |  |
| 319 | BP5_CDS_2926 | shell | hypothetical protein | BP5_CDS_2926 | BVE_02955 | 1 |  |  |  |  |  |  |  |
| 320 | BP5_CDS_2927 | shell | hypothetical protein | BP5_CDS_2927 | BVE_02956 | 1 |  |  |  |  |  |  |  |
| 321 | BP5_CDS_2928 | shell | hypothetical protein | BP5_CDS_2928 | BVE_02957 | 1 |  |  |  |  |  |  |  |
| 322 | BP5_CDS_2929 | shell | hypothetical protein | BP5_CDS_2929 | BVE_02958 | 1 |  |  |  |  |  |  |  |
| 323 | BP5_CDS_2930 | shell | hypothetical protein | BP5_CDS_2930 | BVE_02959 | 1 |  |  |  |  |  |  |  |
| 324 | BP5_CDS_2931 | shell | hypothetical protein | BP5_CDS_2931 | BVE_02960 | 1 |  |  |  |  |  |  |  |
| 325 | BP5_CDS_2932 | shell | hypothetical protein | BP5_CDS_2932 | BVE_02961 | 1 |  |  |  |  |  |  |  |
| 326 | BP5_CDS_2933 | shell | hypothetical protein | BP5_CDS_2933 | BVE_02962 | 1 |  |  |  |  |  |  |  |
| 327 | BP5_CDS_2934 | shell | hypothetical protein | BP5_CDS_2934 | BVE_02963 | 1 |  |  |  |  |  |  |  |
| 328 | BP5_CDS_2936 | shell | hypothetical protein | BP5_CDS_2936 | BVE_02965 | 1 |  |  |  |  |  |  |  |
| 329 | BP5_CDS_2937 | shell | hypothetical protein | BP5_CDS_2937 | BVE_02966 | 1 |  |  |  |  |  |  |  |
| 330 | BP5_CDS_2938 | shell | hypothetical protein | BP5_CDS_2938 | BVE_02967 | 1 |  |  |  |  |  |  |  |
| 331 | BP5_CDS_2939 | shell | hypothetical protein | BP5_CDS_2939 | BVE_02968 | 1 |  |  |  |  |  |  |  |
| 332 | BP5_CDS_2940 | shell | hypothetical protein | BP5_CDS_2940 | BVE_02969 | 1 |  |  |  |  |  |  |  |
| 333 | BP5_CDS_2941 | shell | hypothetical protein | BP5_CDS_2941 | BVE_02970 | 1 |  |  |  |  |  |  |  |
| 334 | BP5_CDS_2942 | shell | hypothetical protein | BP5_CDS_2942 | BVE_02971 | 1 |  |  |  |  |  |  |  |
| 335 | BP5_CDS_2944 | shell | hypothetical protein | BP5_CDS_2944 | BVE_02973 | 1 |  |  |  |  |  |  |  |
| 336 | BP5_CDS_2945 | shell | hypothetical protein | BP5_CDS_2945 | BVE_02974 | 1 |  |  |  |  |  |  |  |
| 337 | BP5_CDS_2946 | shell | hypothetical protein | BP5_CDS_2946 | BVE_02975 | 1 |  |  |  |  |  |  |  |
| 338 | BP5_CDS_2947 | shell | hypothetical protein | BP5_CDS_2947 | BVE_02976 | 1 |  |  |  |  |  |  |  |
| 339 | BP5_CDS_2948 | shell | hypothetical protein | BP5_CDS_2948 | BVE_02977 | 1 |  |  |  |  |  |  |  |
| 340 | BP5_CDS_2949 | shell | hypothetical protein | BP5_CDS_2949 | BVE_02978 | 1 |  |  |  |  |  |  |  |
| 341 | BP5_CDS_2950 | shell | hypothetical protein | BP5_CDS_2950 | BVE_02979 | 1 |  |  |  |  |  |  |  |
| 342 | BP5_CDS_2951 | shell | hypothetical protein | BP5_CDS_2951 | BVE_02980 | 1 |  |  |  |  |  |  |  |
| 343 | BP5_CDS_2952 | shell | hypothetical protein | BP5_CDS_2952 | BVE_02981 | 1 |  |  |  |  |  |  |  |
| 344 | BP5_CDS_2953 | shell | hypothetical protein | BP5_CDS_2953 | BVE_02982 | 1 |  |  |  |  |  |  |  |
| 345 | BP5_CDS_2954 | shell | hypothetical protein | BP5_CDS_2954 | BVE_02983 | 1 |  |  |  |  |  |  |  |
| 346 | BP5_CDS_2955 | shell | hypothetical protein | BP5_CDS_2955 | BVE_02984 | 1 |  |  |  |  |  |  |  |
| 347 | BP5_CDS_2956 | shell | hypothetical protein | BP5_CDS_2956 | BVE_02985 | 1 |  |  |  |  |  |  |  |
| 348 | BP5_CDS_2957 | shell | hypothetical protein | BP5_CDS_2957 | BVE_02986 | 1 |  |  |  |  |  |  |  |
| 349 | BP5_CDS_2958 | shell | hypothetical protein | BP5_CDS_2958 | BVE_02987 | 1 |  |  |  |  |  |  |  |
| 350 | BP5_CDS_2959 | shell | hypothetical protein | BP5_CDS_2959 | BVE_02988 | 1 |  |  |  |  |  |  |  |
| 351 | BP5_CDS_2960 | shell | hypothetical protein | BP5_CDS_2960 | BVE_02989 | 1 |  |  |  |  |  |  |  |
| 352 | BP5_CDS_2961 | shell | hypothetical protein | BP5_CDS_2961 | BVE_02990 | 1 |  |  |  |  |  |  |  |
| 353 | BP5_CDS_2962 | shell | hypothetical protein | BP5_CDS_2962 | BVE_02991 | 1 |  |  |  |  |  |  |  |
| 354 | BP5_CDS_2963 | shell | hypothetical protein | BP5_CDS_2963 | BVE_02992 | 1 |  |  |  |  |  |  |  |
| 355 | BP5_CDS_2964 | shell | hypothetical protein | BP5_CDS_2964 | BVE_02993 | 1 |  |  |  |  |  |  |  |
| 356 | BP5_CDS_2965 | shell | hypothetical protein | BP5_CDS_2965 | BVE_02994 | 1 |  |  |  |  |  |  |  |
| 357 | BP5_CDS_2966 | shell | hypothetical protein | BP5_CDS_2966 | BVE_02995 | 1 |  |  |  |  |  |  |  |
| 358 | BP5_CDS_2967 | shell | hypothetical protein | BP5_CDS_2967 | BVE_02996 | 1 |  |  |  |  |  |  |  |
| 359 | BP5_CDS_2968 | shell | hypothetical protein | BP5_CDS_2968 | BVE_02997 | 1 |  |  |  |  |  |  |  |
| 360 | BP5_CDS_2969 | shell | hypothetical protein | BP5_CDS_2969 | BVE_02998 | 1 |  |  |  |  |  |  |  |
| 361 | BP5_CDS_2970 | shell | hypothetical protein | BP5_CDS_2970 | BVE_02999 | 1 |  |  |  |  |  |  |  |
| 362 | BP5_CDS_2971 | shell | hypothetical protein | BP5_CDS_2971 | BVE_03000 | 1 |  |  |  |  |  |  |  |
| 363 | BP5_CDS_2972 | shell | hypothetical protein | BP5_CDS_2972 | BVE_03001 | 1 |  |  |  |  |  |  |  |
| 364 | BP5_CDS_2973 | shell | hypothetical protein | BP5_CDS_2973 | BVE_03002 | 1 |  |  |  |  |  |  |  |
| 365 | BP5_CDS_2974 | shell | hypothetical protein | BP5_CDS_2974 | BVE_03003 | 1 |  |  |  |  |  |  |  |
| 366 | BP5_CDS_2975 | shell | hypothetical protein | BP5_CDS_2975 | BVE_03004 | 1 |  |  |  |  |  |  |  |
| 367 | BP5_CDS_2976 | shell | hypothetical protein | BP5_CDS_2976 | BVE_03005 | 1 |  |  |  |  |  |  |  |
| 368 | BP5_CDS_2977 | shell | hypothetical protein | BP5_CDS_2977 | BVE_03006 | 1 |  |  |  |  |  |  |  |
| 369 | BP5_CDS_2979 | shell | hypothetical protein | BP5_CDS_2979 | BVE_03008 | 1 |  |  |  |  |  |  |  |
| 370 | BP5_CDS_2980 | shell | hypothetical protein | BP5_CDS_2980 | BVE_03009 | 1 |  |  |  |  |  |  |  |
| 371 | BP5_CDS_2981 | shell | hypothetical protein | BP5_CDS_2981 | BVE_03010 | 1 |  |  |  |  |  |  |  |
| 372 | BP5_CDS_2982 | shell | hypothetical protein | BP5_CDS_2982 | BVE_03011 | 1 |  |  |  |  |  |  |  |
| 373 | BP5_CDS_2983 | shell | hypothetical protein | BP5_CDS_2983 | BVE_03012 | 1 |  |  |  |  |  |  |  |
| 374 | BP5_CDS_2984 | shell | hypothetical protein | BP5_CDS_2984 | BVE_03013 | 1 |  |  |  |  |  |  |  |
| 375 | BP5_CDS_2986 | shell | hypothetical protein | BP5_CDS_2986 | BVE_03015 | 1 |  |  |  |  |  |  |  |
| 376 | BP5_CDS_2987 | shell | hypothetical protein | BP5_CDS_2987 | BVE_03016 | 1 |  |  |  |  |  |  |  |
| 377 | BP5_CDS_2988 | shell | hypothetical protein | BP5_CDS_2988 | BVE_03017 | 1 |  |  |  |  |  |  |  |
| 378 | BP5_CDS_2989 | shell | hypothetical protein | BP5_CDS_2989 | BVE_03018 | 1 |  |  |  |  |  |  |  |
| 379 | BP5_CDS_2990 | shell | hypothetical protein | BP5_CDS_2990 | BVE_03019 | 1 |  |  |  |  |  |  |  |
| 380 | BP5_CDS_2991 | shell | hypothetical protein | BP5_CDS_2991 | BVE_03020 | 1 |  |  |  |  |  |  |  |
| 381 | BP5_CDS_2992 | shell | hypothetical protein | BP5_CDS_2992 | BVE_03021 | 1 |  |  |  |  |  |  |  |
| 382 | BP5_CDS_2993 | shell | hypothetical protein | BP5_CDS_2993 | BVE_03022 | 1 |  |  |  |  |  |  |  |
| 383 | BP5_CDS_2994 | shell | hypothetical protein | BP5_CDS_2994 | BVE_03023 | 1 |  |  |  |  |  |  |  |
| 384 | BP5_CDS_2995 | shell | hypothetical protein | BP5_CDS_2995 | BVE_03024 | 1 |  |  |  |  |  |  |  |
| 385 | BP5_CDS_2996 | shell | hypothetical protein | BP5_CDS_2996 | BVE_03025 | 1 |  |  |  |  |  |  |  |
| 386 | BP5_CDS_2997 | shell | hypothetical protein | BP5_CDS_2997 | BVE_03026 | 1 |  |  |  |  |  |  |  |
| 387 | BP5_CDS_2998 | shell | hypothetical protein | BP5_CDS_2998 | BVE_03027 | 1 |  |  |  |  |  |  |  |
| 388 | BP5_CDS_2999 | shell | hypothetical protein | BP5_CDS_2999 | BVE_03028 | 1 |  |  |  |  |  |  |  |
| 389 | BP5_CDS_3000 | shell | hypothetical protein | BP5_CDS_3000 | BVE_03029 | 1 |  |  |  |  |  |  |  |
| 390 | BP5_CDS_3001 | shell | hypothetical protein | BP5_CDS_3001 | BVE_03030 | 1 |  |  |  |  |  |  |  |
| 391 | BP5_CDS_3002 | shell | hypothetical protein | BP5_CDS_3002 | BVE_03031 | 1 |  |  |  |  |  |  |  |
| 392 | BP5_CDS_3003 | shell | hypothetical protein | BP5_CDS_3003 | BVE_03032 | 1 |  |  |  |  |  |  |  |
| 393 | BP5_CDS_3004 | shell | hypothetical protein | BP5_CDS_3004 | BVE_03033 | 1 |  |  |  |  |  |  |  |
| 394 | BP5_CDS_3005 | shell | hypothetical protein | BP5_CDS_3005 | BVE_03034 | 1 |  |  |  |  |  |  |  |
| 395 | BP5_CDS_3006 | shell | hypothetical protein | BP5_CDS_3006 | BVE_03035 | 1 |  |  |  |  |  |  |  |
| 396 | BP5_CDS_3007 | shell | hypothetical protein | BP5_CDS_3007 | BVE_03036 | 1 |  |  |  |  |  |  |  |
| 397 | BP5_CDS_3008 | shell | hypothetical protein | BP5_CDS_3008 | BVE_03037 | 1 |  |  |  |  |  |  |  |
| 398 | BP5_CDS_3010 | shell | hypothetical protein | BP5_CDS_3010 | BVE_03039 | 1 |  |  |  |  |  |  |  |
| 399 | BP5_CDS_3011 | shell | hypothetical protein | BP5_CDS_3011 | BVE_03040 | 1 |  |  |  |  |  |  |  |
| 400 | BP5_CDS_3012 | shell | hypothetical protein | BP5_CDS_3012 | BVE_03041 | 1 |  |  |  |  |  |  |  |
| 401 | BP5_CDS_3013 | shell | hypothetical protein | BP5_CDS_3013 | BVE_03042 | 1 |  |  |  |  |  |  |  |
| 402 | BP5_CDS_3107 | persistent | hypothetical protein | BP5_CDS_3107 | BVE_03136 | 1 |  |  |  |  |  |  |  |
| 403 | BP5_CDS_3115 | persistent | hypothetical protein | BP5_CDS_3115 | BVE_03144 | 1 |  |  |  |  |  |  |  |
| 404 | BP5_CDS_3166 | shell | hypothetical protein | BP5_CDS_3166 | BVE_03202 | 1 |  |  |  |  |  |  |  |
| 405 | BP5_CDS_3173 | persistent | hypothetical protein | BP5_CDS_3173 | BVE_03209 | 1 |  |  |  |  |  |  |  |
| 406 | BP5_CDS_3176 | persistent | hypothetical protein | BP5_CDS_3176 | BVE_03212 | 1 |  |  |  |  |  |  |  |
| 407 | BP5_CDS_3179 | shell | hypothetical protein | BP5_CDS_3179 | BVE_03215 | 1 |  |  |  |  |  |  |  |
| 408 | BP5_CDS_3180 | shell | hypothetical protein | BP5_CDS_3180 | BVE_03216 | 1 |  |  |  |  |  |  |  |
| 409 | BP5_CDS_3181 | shell | hypothetical protein | BP5_CDS_3181 | BVE_03217 | 1 |  |  |  |  |  |  |  |
| 410 | BP5_CDS_3183 | shell | hypothetical protein | BP5_CDS_3183 | BVE_03219 | 1 |  |  |  |  |  |  |  |
| 411 | BP5_CDS_3218 | persistent | hypothetical protein | BP5_CDS_3218 | BVE_03254 | 1 |  |  |  |  |  |  |  |
| 412 | BP5_CDS_3219 | persistent | hypothetical protein | BP5_CDS_3219 | BVE_03255 | 1 |  |  |  |  |  |  |  |
| 413 | BP5_CDS_3408 | persistent | hypothetical protein | BP5_CDS_3408 | BVE_03446 | 1 |  |  |  |  |  |  |  |
| 414 | BP5_CDS_3419 | persistent | hypothetical protein | BP5_CDS_3419 | BVE_03457 | 1 |  |  |  |  |  |  |  |
| 415 | BP5_CDS_3422 | shell | hypothetical protein | BP5_CDS_3422 | BVE_03460 | 1 |  |  |  |  |  |  |  |
| 416 | BP5_CDS_3424 | shell | hypothetical protein | BP5_CDS_3424 | BVE_03462 | 1 |  |  |  |  |  |  |  |
| 417 | BP5_CDS_3425 | shell | hypothetical protein | BP5_CDS_3425 | BVE_03463 | 1 |  |  |  |  |  |  |  |
| 418 | BP5_CDS_3427 | shell | hypothetical protein | BP5_CDS_3427 | BVE_03465 | 1 |  |  |  |  |  |  |  |
| 419 | BP5_CDS_3429 | shell | hypothetical protein | BP5_CDS_3429 | BVE_03467 | 1 |  |  |  |  |  |  |  |
| 420 | BP5_CDS_3431 | shell | hypothetical protein | BP5_CDS_3431 | BVE_03469 | 1 |  |  |  |  |  |  |  |
| 421 | BP5_CDS_3432 | shell | hypothetical protein | BP5_CDS_3432 | BVE_03470 | 1 |  |  |  |  |  |  |  |
| 422 | BP5_CDS_3433 | shell | hypothetical protein | BP5_CDS_3433 | BVE_03471 | 1 |  |  |  |  |  |  |  |
| 423 | BP5_CDS_3434 | shell | hypothetical protein | BP5_CDS_3434 | BVE_03472 | 1 |  |  |  |  |  |  |  |
| 424 | BP5_CDS_3436 | shell | hypothetical protein | BP5_CDS_3436 | BVE_03474 | 1 |  |  |  |  |  |  |  |
| 425 | BP5_CDS_3437 | shell | hypothetical protein | BP5_CDS_3437 | BVE_03475 | 1 |  |  |  |  |  |  |  |
| 426 | BP5_CDS_3438 | shell | hypothetical protein | BP5_CDS_3438 | BVE_03476 | 1 |  |  |  |  |  |  |  |
| 427 | BP5_CDS_3439 | shell | hypothetical protein | BP5_CDS_3439 | BVE_03477 | 1 |  |  |  |  |  |  |  |
| 428 | BP5_CDS_3440 | shell | hypothetical protein | BP5_CDS_1070;  BP5_CDS_3440 | BVE_01078;  BVE_03478 | 2 |  |  |  |  |  |  |  |
| 429 | BP5_CDS_3441 | shell | hypothetical protein | BP5_CDS_3441 | BVE_03479 | 1 |  |  |  |  |  |  |  |
| 430 | BP5_CDS_3442 | shell | hypothetical protein | BP5_CDS_3442 | BVE_03480 | 1 |  |  |  |  |  |  |  |
| 431 | BP5_CDS_3539 | persistent | hypothetical protein | BP5_CDS_3539 | BVE_03577 | 1 |  |  |  |  |  |  |  |
| 432 | BP5_CDS_3596 | persistent | hypothetical protein | BP5_CDS_3596 | BVE_03639 | 1 |  |  |  |  |  |  |  |
| 433 | BP5_CDS_3625 | persistent | hypothetical protein | BP5_CDS_3625 | BVE_03668 | 1 |  |  |  |  |  |  |  |
| 434 | BP5_CDS_3646 | shell | hypothetical protein | BP5_CDS_3646 | BVE_03689 | 1 |  |  |  |  |  |  |  |
| 435 | BP5_CDS_3648 | shell | hypothetical protein | BP5_CDS_3648 | BVE_03691 | 1 |  |  |  |  |  |  |  |
| 436 | BP5_CDS_3725 | persistent | hypothetical protein | BP5_CDS_3725 | BVE_03770 | 1 |  |  |  |  |  |  |  |
| 437 | BP5_CDS_3730 | shell | hypothetical protein | BP5_CDS_3730 | BVE_03775 | 1 |  |  |  |  |  |  |  |
| 438 | BP5_CDS_3731 | shell | hypothetical protein | BP5_CDS_3731 | BVE_03776 | 1 |  |  |  |  |  |  |  |
| 439 | BP5_CDS_3780 | persistent | hypothetical protein | BP5_CDS_3780 | BVE_03825 | 1 |  |  |  |  |  |  |  |
| 440 | BP5_CDS_3993 | shell | hypothetical protein | BP5_CDS_3993 | BVE_04041 | 1 |  |  |  |  |  |  |  |
| 441 | BP5_CDS_3994 | shell | hypothetical protein | BP5_CDS_3994 | BVE_04042 | 1 |  |  |  |  |  |  |  |
| 442 | BP5_CDS_3997 | shell | hypothetical protein | BP5_CDS_3997 | BVE_04045 | 1 |  |  |  |  |  |  |  |
| 443 | BP5_CDS_3998 | shell | hypothetical protein | BP5_CDS_3998 | BVE_04061 | 1 |  |  |  |  |  |  |  |
| 444 | BP5_CDS_0277 | shell | putative ABC transporter ATP-binding protein YknY | BP5_CDS_0277 | BVE_00282 | 1 |  |  |  |  |  |  |  |
| 445 | BP5_CDS_1343 | shell | putative ABC transporter ATP-binding protein YxlF | BP5_CDS_1343 | BVE_01351 | 1 |  |  |  |  |  |  |  |
| 446 | BP5_CDS_3643 | shell | putative antitoxin YezG | BP5_CDS_3643 | BVE_03686 | 1 |  |  |  |  |  |  |  |
| 447 | BP5_CDS_3644 | shell | putative antitoxin YezG | BP5_CDS_3644 | BVE_03687 | 1 |  |  |  |  |  |  |  |
| 448 | BP5_CDS_3645 | shell | putative antitoxin YezG | BP5_CDS_3645 | BVE_03688 | 1 |  |  |  |  |  |  |  |
| 449 | BP5_CDS_0355 | shell | putative beta-barrel protein YwiB | BP5_CDS_0355 | BVE_00360 | 1 |  |  |  |  |  |  |  |
| 450 | BP5_CDS_3216 | persistent | putative methyltransferase YcgJ | BP5_CDS_3216 | BVE_03252 | 1 |  |  |  |  |  |  |  |
| 451 | BP5_CDS_2908 | shell | putative protein YobA | BP5_CDS_2908 | BVE_02937 | 1 |  |  |  |  |  |  |  |
| 452 | BP5_CDS_1005 | shell | putative protein YqaI | BP5_CDS_1005 | BVE_01013 | 1 |  |  |  |  |  |  |  |
| 453 | BP5_CDS_1041 | shell | putative protein YqbO | BP5_CDS_1041 | BVE_01049 | 1 |  |  |  |  |  |  |  |
| 454 | BP5_CDS_1051 | shell | putative ribonuclease YokI | BP5_CDS_1051 | BVE_01059 | 1 |  |  |  |  |  |  |  |
| 455 | BP5_CDS_3423 | shell | putative ribonuclease YokI | BP5_CDS_3423 | BVE_03461 | 1 |  |  |  |  |  |  |  |
| 456 | BP5_CDS_0177 | shell | tRNA nuclease WapA | BP5_CDS_0177 | BVE_00182 | 1 |  |  |  |  |  |  |  |

**Table S6.** Comparative analysis of secondary metabolite gene clusters among *B. velezensis* BP5, BP103.

| **Gene** | **Type** | **Size** | **Most similar** | **Bioactivity** | **Contig position (From-To)** | |
| --- | --- | --- | --- | --- | --- | --- |
|  |  |  |  |  | **BP103** | **BP5** |
| 1 | NRPS, betalactone | ~87,792 | Fengycin | Antibacterial and antifungal | contig_1: 1-88,738 | contig_4: 1-87,792 |
| 2 | TransAT-PKS + NRPS + NRPS-like + T3PKS | ~110,124 | Bacillaene | Antibacterial and antifungal | contig_1: 154,433-264,556 | contig_4: 143,016-253,136 |
| 3 | TransAT-PKS | 88.201 | Macrolactin H | Antibacterial | contig_1: 486,655-574,855 | contig_2: 23,074-111,274 |
| 4 | Terpene | ~20,740 | -a | -a | contig_1: 872,567-893,307 | contig_2: 424,967-445,707 |
| 5 | PKS-like | 41.245 | - | - | contig_1: 975,356-1,016,600 | contig_2: 527,733-568,977 |
| 6 | Terpene-precursor, NRP-metallophore, NRPS, RiPP | 65.361 | Bacillibactin | iron-acquisition, and antibacterial | contig_2: 63,615-128,975 | contig_1: 876,065-941,425 |
| 7 | Other | 41.419 | Bacilysin | Antibacterial | contig_2: 658,352-699,770 | contig_1: 304,897-346,315 |
| 8 | Lanthipeptide-class-II | ~23,189 | Mersacidin | Antibacterial | contig_2: 835,824-859,012 | contig_2: 275,122-302,011 |
| 9 | T3PKS | 41.101 | – | – | contig_3: 172,282-213,382 | contig_5: 134,676-175,776 |
| 10 | TransAT-PKS | 106.167 | Difficidin | Antibacterial | contig_3: 328,819-434,985 | contig_3: 1-44,054 |
|  |  |  |  |  |  | contig_13: 1-52,894 |
| 11 | Terpene-precursor | 20.891 | – | – | contig_3: 458,313-479,203 | contig_3: 57,372-78,262 |
| 12 | NRPS | ~65,411 | Surfactin | Antibacterial | contig_4: 194,410-259,820 | contig_6: 1-25,183 |
|  |  |  |  |  |  | contig_8: 1-27,680 |
| 13 | NRPS + transAT-PKS | ~77,727 | Locillomycin (B/C) | Antifungal | contig_4: 332,188-409,914 | contig_8: 97,757-175,485 |

**Table S7.** The number of gene families across the genomes of *Bacillus* spp. BP5 and BP103 and related strains

| **Strain** | **GH** | **GT** | **CE** | **PL** | **AA** | **CBM** | **Total CAZymes** |
| --- | --- | --- | --- | --- | --- | --- | --- |
| *Bacillus velezensis* 77-E7 | 45 | 42 | 13 | 3 | 4 | 31 | 138 |
| *Bacillus velezensis* CBMB205 | 40 | 40 | 14 | 3 | 5 | 28 | 130 |
| *Bacillus subtilis* DSM10 | 57 | 44 | 15 | 7 | 4 | 40 | 167 |
| *Bacillus velezensis* FZB42 | 40 | 40 | 14 | 3 | 5 | 27 | 129 |
| *Bacillus velezensis* BP103 | 40 | 40 | 13 | 3 | 5 | 28 | 129 |
| *Bacillus velezensis* BP5 | 42 | 40 | 14 | 3 | 5 | 29 | 133 |

*Note:*

*GH (Glycoside Hydrolases)*

*GT (GlycosylTransferases)*

*CE (Carbohydrate Esterases)*

*PL (Polysaccharide Lyases)*

*AA (Auxiliary Activities)*

*CBM (Carbohydrate-Binding Modules)*
